# Anoikis: To Die or Not to Die?

**DOI:** 10.3390/ijms27020579

**Published:** 2026-01-06

**Authors:** Tomas Koltai, Larry Fliegel

**Affiliations:** 1 Hospital del Centro Gallego de Buenos Aires, Buenos Aires 2199, Argentina; 2 Department of Biochemistry, University Alberta, Edmonton, AB T6G 2H7, Canada

**Keywords:** anoikis, apoptosis, cancer, epithelial–mesenchymal transition, focal adhesion, integrins, metastasis

## Abstract

Epithelial, endothelial, and many connective tissue cells are normally attached to the extracellular matrix (ECM). These cells rely on the ECM for structural support, signaling, and regulation of their behavior. When these cells lose this attachment or are in an inappropriate location, these cells soon die by a mechanism called anoikis (homelessness). Anoikis is a programmed cell death of an apoptotic nature; however, it can, in certain cases, be overcome, and detached cells can survive in the absence of the correct signals from the ECM. This is the case of malignant cells, where anoikis resistance is a prerequisite for invasion and metastasis. Without anoikis resistance (anchorage-independency), tumors would be unable to abandon their normal sites and would invade neighboring tissues and metastasize at distant locations. Anoikis is the natural barrier against cancer progression. Therefore, overcoming anoikis is a major step in cellular transformation. Cancer cells have developed many successful strategies to bypass anoikis. The main mechanism, albeit not the only one, involves hyper-activating survival pathways and over-expressing anti-apoptotic molecules. There is a strong and intertwining association between epithelial–mesenchymal transition and anoikis resistance that is discussed in depth. A better understanding of these anoikis resistance mechanisms has led to the research and development of pharmaceuticals that can counteract them.

## 1. Introduction

### 1.1. Anoikis

Normal tissues have a delicate dynamic balance of cell proliferation and cell death that forms part of an essential homeostasis of multicellular organisms. Anoikis contributes to this homeostasis. Anoikis (meaning homelessness in Greek) is defined as a programmed cell death that is induced upon cell detachment from the extracellular matrix (ECM). Anoikis is also known as cell-detachment-induced apoptosis. Sakamoto and Kyprianou [1] described this phenomenon clearly and succinctly: cells gain freedom and meet death. In laymen’s terms, it could be restated as a form of apoptosis that occurs when cells are detached from the extracellular matrix. Those cells that lose their established permanent contacts with the extracellular matrix are bound to die. The words “established permanent” differentiate cells that have permanent contact with their ECM, such as epithelial, endothelial, osteoblasts, chondrocytes, and even some connective tissue cells, from those that can have occasional, but not usually firmly established, contact, such as circulating leucocytes, dendritic cells, and macrophages. Therefore, we can differentiate attachment-dependent cells like epithelial cells and others from attachment-independent cells such as white blood cells. Attachment-dependent cells “need” to be attached to the ECM to survive and thrive, while attachment-independent cells do not. Solid tumors are characterized by the transformation of attachment-dependent to attachment-independent cells. This transformation implies different pathways and molecules that will be analyzed in this review.

Normal attachment-dependent cells have a permanent crosstalk with the ECM to which they are attached. This crosstalk is essential for their survival because it influences cell shape, migration, proliferation, and differentiation [2]. The ECM is not just a scaffold—it is a dynamic environment that communicates with cells to regulate their fate and function. When cell–ECM communication is interrupted, these cells die. This is called anoikis. On the other hand, cancer cells manage to survive after the interruption of the crosstalk, meaning that they became attachment-independent. Attachment-independent growth is a hallmark of transformation in cancer biology, often used as a test for tumorigenicity in vitro [3].

### 1.2. Resistance to Anoikis Introduction

Resistance to anoikis, a very important hallmark of cancer, consists of a cancer cell’s survival despite its detachment from the ECM. Resistance to anoikis (AR), or cell survival despite detachment, is the loss of the natural barrier that prevents invasion and metastasis. Among the many changes that circulating cancer cells develop, one of the most essential is anoikis resistance. Cancer cells that resist anoikis can survive in suspension, circulate through the bloodstream, and seed metastases [4]. Therefore, while anoikis contributes to the death of circulating tumor cells that detach from primary tumors preventing them from colonizing distant organs, AR has the opposite effects, enhancing colonization and metastatic potential [5]. Furthermore, AR generally correlates with poor prognosis. This has therapeutic implications, because targeting AR mechanisms may lead to the following:

- Prevention of metastasis;
- Sensitization of circulating cancer cells to apoptosis;
- Improvement in outcomes in advanced cancers.

### 1.3. Cell–Matrix Interactions

Cell–matrix interactions have profound effects on phenotype and gene expression and govern several aspects of cell growth, differentiation, and motility [6]. Attachment-dependent cells can survive for a very short time when they lose their attachment and finally die. This means that by losing their attachment, they are not viable anymore.

Cancer cells, on the other hand, can survive when they are detached from their ECM. Multiple mechanisms are developed in the cancer cell to achieve an attachment-independent state. Developing this independence is an event that precedes mobility, invasion, and metastasis.

The millions of viable circulating cancer cells found in most tumors would not be possible without acquiring an attachment-independent status. This is a pre-condition for invasion and metastasis [7].

Crosstalk between the extracellular matrix (ECM) and malignant cells plays a pivotal role in promoting anoikis resistance, enabling cancer cells to survive detachment and metastasize. This interaction reprograms signaling and metabolic pathways that normally trigger cell death upon loss of adhesion.

## 2. Historical Background

In 1968, Stoker et al. [8] found that “*Many cell types will grow when attached to a rigid surface but not in suspension, a phenomenon termed “anchorage dependence*”. Further: “*The state of inhibited cells in suspension culture was examined by dispersing them in a methyl cellulose gel, in vessels lined with agar. In this system aggregation is prevented and the cells may be recovered quantitatively. Normal, as well as transformed, cells increase in size, and a proportion synthetize DNA during the first 24 h in suspension culture. Growth and DNA synthesis in normal cells then virtually cease, while transformed cells continue to grow into colonies.*”

Based on these previous experiments, anoikis was described for the first time in 1994 by Frisch and Francis [9]. They also coined the name. Furthermore, in the same article, they showed that transformed cells were resistant to anoikis and that there were attachment-dependent cells such as the epithelial cells and attachment-independent cells like fibroblasts. In that single article they established the pillars on which the anoikis building was erected. Importantly, they clearly showed the essential relationship between the ECM and cells and that apoptosis could be regulated by the ECM. The origin of the Frisch and Francis studies stemmed from their idea that “*cell motility or transformation might require matrix-independent survival*”.

## 3. Anoikis Concept

Under physiological conditions, anoikis maintains cell number balance by triggering apoptosis in cells with inadequate cell-to-ECM and cell-to-cell interactions [10]. A good example of anoikis is the death of intestinal epithelial cells that are detached and shed into the lumen and undergo apoptosis [11]. Anoikis is found in many normal epithelial cells that permanently or periodically renew their cell portfolio. This includes mammary gland [12,13], thyroid cells, intestinal mucosa, keratinocytes, and other cell types [14]. Anoikis is also present in normal endothelial cells and osteoclasts [15].

Many publications maintain that anoikis is a mechanism for preventing adherent-independent cell growth and attachment to an inappropriate matrix, thus avoiding colonization of distant organs. This would mean that the cell commits this suicidal act because it “knows” it is dangerous to allow a cell to circulate out of control. We do not agree with this concept. It is hard to believe that individual cells are conscious of the danger they represent. We believe that what drives anoikis is not the cell’s “knowledge”. What drives it is the loss of stimulatory and regulatory signals received from the extracellular matrix and fellow cells of the epithelium. Anoikis occurs “because of something” rather than “for something”. This does not mean that anoikis is purposeless.

Anoikis is an evolutionary trait developed in a Darwinian fashion in multicellular organisms. Its development confers selective advantages such as preventing cell growth in unusual sites, invasion among tissues, epithelial–mesenchymal transition, and metastasis. It would thus be selected for evolutionarily. At a certain point, evolution from a unicellular to a multicellular organism requires the ability to only grow and differentiate when the cell is in the correct place in a tissue. Otherwise, by the process of anoikis, a cell removes itself through apoptosis. However, we could not find any publications on the phylogenetic evolution of anoikis. Detection of “being in the right place” means that the cell has developed sensing mechanisms that inform it where it is. In addition, these sensing mechanisms have the ability to trigger death pathways if the place is not the correct one.

Anoikis also means that cells have intercommunication with the matrix and other cells in order to recognize their place. In this regard, Gilmore [16] wrote “Anoikis, therefore, should not be considered as an experimental system in vitro, but the mechanism by which cells in vivo use ECM-derived signals to maintain tissue integrity”. Here, we must add that cells need to recognize their correct extracellular site as well as being with their neighbor cells.

To understand anoikis, it is necessary to study the main interaction point between the ECM and the cell, that is, focal adhesion. Focal adhesions are the origin of the signaling that keeps the cell alive while attached to the ECM and initiates apoptosis when detached.

In summary: Attachment-dependent cells die when detached: anoikis; cancer cells do not die when detached: resistance to anoikis; attached normal cells permanently receive signals from the ECM, mainly through integrins, and detachment interrupts these signals, triggering anoikis. When cells lose their attachment to the ECM, the cell cycle is interrupted, and a caspase-dependent cell death occurs: anoikis. Focal adhesions are hubs that permit cell-to-matrix adhesion and at the same time transmit signals from the ECM to the cell and vice versa. ECM detachment in tumors precedes abnormal dissemination that leads to metastasis.

## 4. Focal Adhesions (FAs)

Focal adhesions are mechanical linkages between the cell and the ECM. These adhesions represent a biochemical signaling and adhesion hub where there is a large concentration of adhesive molecules such as integrins. Focal adhesions are very dynamic and are made up of more than 100 different proteins that are in a constant state of change. Focal adhesions form following the cell’s attachment to the ECM through integrins, anchoring actin filaments and microtubules (MTs) to the cell’s membrane [17]. Two kinases, FAK (focal adhesion kinase) and SRC, and a protein, Paxillin, are key elements that connect integrins to the actin cytoskeleton through talin and vinculin, two other important proteins.

Some proteins of focal adhesions associate while others disassociate continuously. This generates signals that are transmitted to the rest of the cell by the ECM–integrin–cell axis. Many cell functions such as cell motility, invasion, cell cycle, apoptosis, or resistance to apoptosis are thus influenced by protein trafficking and modifications at the focal adhesion. The large number of different proteins forming these structures may suggest that not all the focal adhesions have the same function. However, there is a lack of information in this respect. The dynamic and permanent changes in the proteins of focal adhesions create the conditions for cell adhesion, survival, growth, differentiation, and motility of normal cells and also invasion, metastasis, and resistance to anoikis in cancer cells.

### 4.1. Focal Adhesions in Cancer

Tumor cell migration and invasion:

FAs facilitate cell motility by coordinating cytoskeleton remodeling and ECM degradation.

Cancer cells exploit FA dynamics to invade surrounding tissues and enter circulation.

Anoikis resistance:

Normally, detachment from the ECM triggers anoikis (a form of apoptosis).

In cancer, FA signaling persists even after detachment, allowing cells to survive and metastasize [18].

Survival and proliferation:

FAs activate key pathways like FAK (focal adhesion kinase), PI3K/AKT, and MAPK/ERK, promoting cell survival and growth.

Mechanotransduction:

FAs help cancer cells adapt to mechanical stress in the tumor microenvironment, enhancing their invasive potential.

### 4.2. Key Molecules Involved in Focal Adhesions

FAK (Focal Adhesion Kinase):

Central to FA signaling; over-expressed in many cancers.

Drives survival, migration, and drug resistance.

Integrins:

Integrins are transmembrane receptors that initiate FA formation.

Altered integrin expression contributes to metastasis and therapy resistance [19].

Changes in integrin profiles enable tumor cells to evade apoptosis, migrate through tissues, and adapt to hostile microenvironments, including during treatment. For example, αvβ3 and α5β1 integrins are associated with poor prognosis and metastatic spread in melanoma, glioblastoma, and colorectal cancer, and integrin β1 is linked to resistance in breast, lung, and pancreatic cancers [20,21,22,23]. Integrins such as β1, αvβ3, and α5β1 bind ECM components and activate survival pathways like FAK/Src, PI3K/AKT, and MAPK/ERK. This signaling cascade inhibits pro-apoptotic factors and promotes cytoskeleton stability, allowing cells to resist detachment-induced apoptosis [24,25].

Adaptor proteins:

Talin, vinculin, paxillin, vimentin, and Src coordinate FA assembly and signal transduction. They are called adaptor proteins because they connect the cytoskeleton to integrins. Although described as adaptor proteins, their functions seem to go beyond adaptation. For example, talin participates in integrin activation [26,27]. Talin over-expression in tumors correlates with resistance to anoikis and metastasis. Furthermore, talin homodimers can bind four integrins, establishing a cross-link of integrins that are essential for clustering [28].

**Figure 1:**
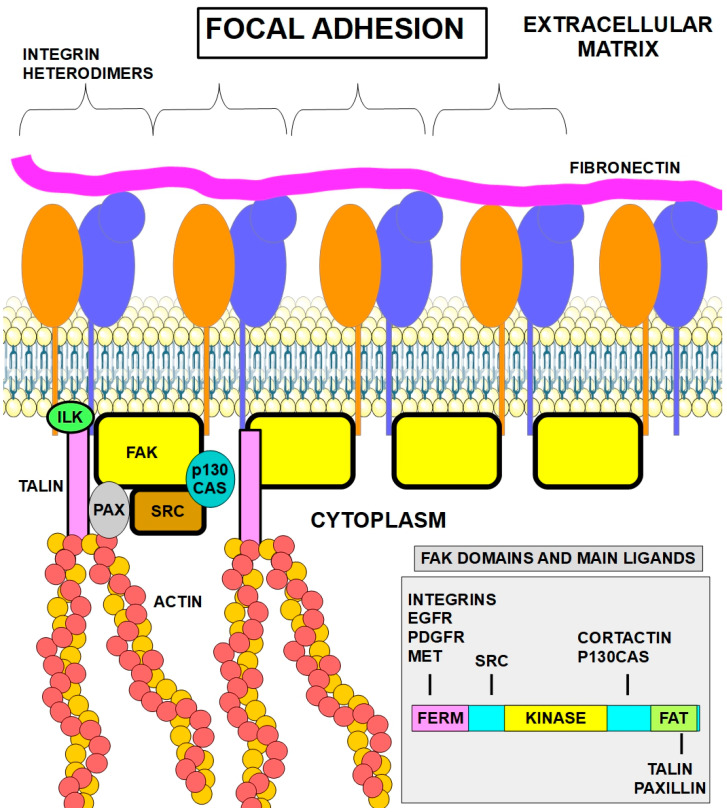
The figure shows the main proteins that interact at the focal adhesion site, where there is a concentration of integrin heterodimers that are attached to extracellular matrix proteins such as fibronectin, collagens, elastin, and laminins. The main attachment of integrins is to fibronectin. This attachment, in addition to its adhesion function, triggers signals to intracellular proteins. The main signaling protein is FAK, which has many binding partners, as shown in the right panel. Autophosphorylation of FAK at tyrosine 397 creates a binding site for the SRC kinase family. The recruitment of SRC further leads to full FAK activation through phosphorylation of other tyrosine residues [29].

Figure 1 shows, in a simplified manner, the main players in the focal adhesion structure. Only a few proteins are represented in the drawing, although they are the most important and best known.

**Figure 2:**
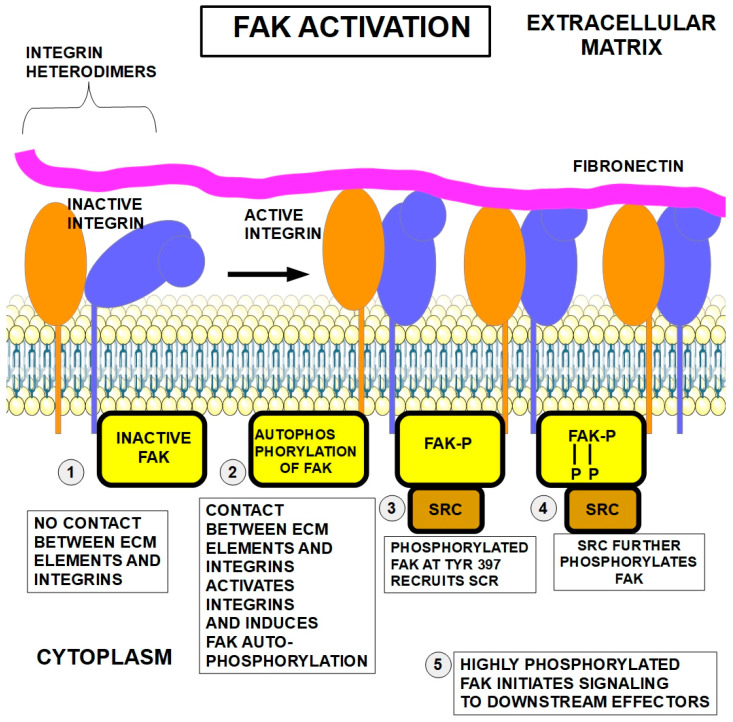
Integrin and FAK activation [30,31,32,33,34] by fibronectin contact with the extracellular portion of the integrin dimer ① and ②. When integrin is not in contact with fibronectin, FAK is inactive ①. Contact between fibronectin and integrin ② causes autophosphorylation of FAK. Phosphorylation of FAK at amino acid Tyr347 recruits SCR ③, which further phosphorylates FAK ④, exerting downstream effects (signaling) ⑤.

The first step of cell adhesion to the ECM consists of contact between fibers (fibronectin, collagens, elastin, and laminins) in the ECM with the extracellular segment of integrin dimers. This step activates integrins that undergo a conformational change (Figure 2). Next, activated integrins, in turn, activate a key protein in the intracellular segment: FAK (focal adhesive kinase). The activation consists of FAK autophosphorylation at tyrosine 397. As mentioned above, talin also plays a role in integrin activation.

In a second step, the phosphorylated FAK recruits and activates another kinase, SRC. SRC continually adds phosphate groups to the many proteins it services. In this case, SRC also adds further phosphates to FAK, permitting full FAK activation (Figure 2④). Now, the highly phosphorylated FAK initiates signaling to downstream effectors.

## 5. The Main Players in Anoikis

### 5.1. Integrins

The central role of integrins in suppressing apoptosis in attached cells through anti-apoptotic and pro-survival signals from the ECM is a well known fact [35]. They transmit outside-in and inside-out signals that regulate survival, proliferation, and migration [14]. Integrins are transmembrane protein adhesive molecules that transmit signals between cells and the extracellular matrix, probably in a bidirectional fashion [36]. Integrins are heterodimers formed by an α and a β subunit that are associated through non-covalent bonding [37]. The integrin family is formed by 24 different heterodimers. Their extracellular ligands make it possible to classify them in four groups:

- RGD-binding integrins: RGD receptors (Arg-Gly-Asp (RGD) attachment site), constitute a major recognition system for cell adhesion [38,39]; several integrins recognize and bind to the RGD motif, a key tripeptide sequence found in many extracellular matrix (ECM) proteins like fibronectin, vitronectin, and fibrinogen. These RGD-binding integrins play crucial roles in cell adhesion, migration, and signaling. Importantly, RGD-binding integrins like αvβ3 and αvβ5 are over-expressed in tumors and promote angiogenesis, invasion, and metastasis [40];
- Laminin receptors that play an important role in cell migration [41,42];
- Leukocyte-specific receptors are a specialized subset of integrins that mediate immune cell adhesion, migration, and signaling. They are essential for immune surveillance, inflammation, and host defense [43]. These integrins are primarily expressed on white blood cells and are often referred to as β2 integrins or CD18 family;
- Collagen receptors that regulate proliferation, migration, and adhesion [44].

Importantly, integrins suppress anoikis [45]. Thus, while an epithelial cell is anchored to the matrix, there is no anoikis. This means that integrin signaling to the cell prevents anoikis-induced apoptosis, but as soon as the cell is detached, this inhibition disappears and the cell dies. If the cell is a malignant one, it does not die because it develops resistance to anoikis.

Integrins have a double function: they anchor the cell to the matrix (adhesive function) and transmit signals between matrix and cell and vice versa (communication function). The precise molecular mechanism of how integrins participate in anoikis and resistance to anoikis is far from clear. What is clear is that anoikis is mediated by membrane-adhesion-signaling molecules such as integrins. Interestingly, integrins can also inhibit anoikis in some detached cells [46].

Integrins as sensors. Integrins act as biochemical and biomechanical sensors, enabling cells to detect and respond to changes in their extracellular environment. This sensing function is crucial for regulating adhesion, migration, survival, and differentiation, especially in cancer and immune responses.

Their sensing capabilities operate through two main modes:

▪ Biochemical Sensing

Ligand recognition: Integrins bind to ECM proteins like fibronectin, collagen, and laminin via specific motifs (e.g., RGD).

Bidirectional signaling [37]: Outside-in signaling: Ligand binding triggers intracellular cascades (e.g., FAK, Src, and PI3K/AKT). Inside-out signaling: cytoplasmic signals modulate integrin affinity and clustering [47,48].

▪ Biomechanical Sensing [49]

There are two important modifications within the ECM that play an essential role in the cancer cell fate: stiffness (rigidity) and degradation [50]. ECM stiffness is frequently found in cancer and plays a role in migration, invasion, and metastasis [51,52]. Importantly, stiffness is detected by cancer cells through their integrin-mediated biomechanical sensing abilities, which elicit intracellular changes that reinforce the malignant phenotype, including resistance to apoptosis. The mechanisms involved are as follows:

Force transmission: Integrins connect the ECM to the actin cytoskeleton, allowing cells to sense mechanical tension.

Mechanotransduction [53]: Integrins convert mechanical stimuli into biochemical signals, influencing cell fate and behavior. Integrins can detect matrix stiffness, leading cells to adjust their adhesion and migration based on ECM rigidity, which is usually altered in cancer.

The integrin properties mentioned above activate various signaling pathways, such as the focal adhesion kinase (FAK) pathway, the Src kinase pathway, and the PI3K/Akt pathway.

However, integrin signaling is not limited to these pathways and can also interact with other signaling molecules, such as growth factor receptors and GPCRs (G-protein-coupled receptors), to modulate their activity.

Integrin antagonists are being developed to block aberrant signaling in cancer. RGD-based drug delivery systems exploit integrins sensing to target tumors with high αvβ3 expression. In this regard, drugs have been developed, such as cilengitide, TDI4161, and MK0469, that target αvβ3 integrins (they are further discussed below).

### 5.2. FAK (Focal Adhesion Kinase)

**Figure 3:**
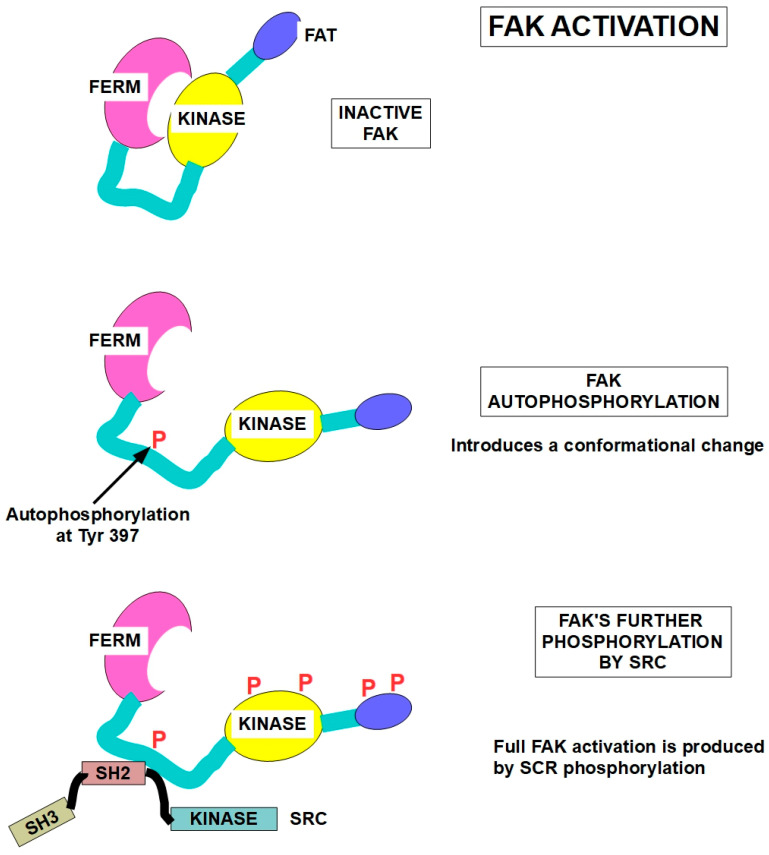
FAK activation [102] and SRC structure [103]. Upper panel: dephosphorylated FAK is inactive. FAK has an N-terminal FERM (For Ezrin, Radixin, and Moesin) domain, a kinase domain, and an FAT (focal-adhesion-targeting) domain. Middle panel: FAK autophosphorylation at Tyr397 induces a conformational change and activates the protein. Lower panel: full activation of FAK at multiple sites is achieved through SRC phosphorylation at several additional sites.

FAK (focal adhesion kinase) is a cytoplasmic protein tyrosine kinase that is activated by phosphorylation when integrins bind to their extracellular ligands such as collagen or fibronectin. Integrins do not have kinase activity, and they do not phosphorylate FAK. They induce FAK’s autophosphorylation. This activation triggers a cascade of signaling events that regulate cell behavior, including cell survival, migration, and proliferation [54,55]. FAK has several functional domains that interact with other proteins at the focal adhesion and can execute various biological processes [56]. FAK has a FERM domain at the N-terminus, a central catalytic kinase domain and a C-terminal focal-adhesion-targeting domain (FAT). (Right lower panel in Figure 3).

FAK acts as a signaling hub, interacting with various proteins such as Src kinases, paxillin, and p130Cas, transmitting downstream signaling, regulating the following:

- Cell survival: it promotes cell survival by inhibiting apoptosis.
- Cell migration: it promotes migration by regulating the assembly and disassembly of focal adhesions [57] and has a role in invadopodia formation [58,59].
- Epithelial–mesenchymal transition is influenced/regulated by FAK-SRC signaling [60].
- Cell proliferation: FAK signaling can promote cell proliferation in response to certain stimuli [61].

FAK is often over-expressed and hyper-active in various types of cancer, such as exocrine pancreas [62,63], non-small-cell lung [64], small-cell lung [65], ovarian [66], gastric [67], colorectal [68], head and neck squamous cell [69], breast [70,71], thyroid [72,73,74], hepatocellular carcinoma [75], clear-cell renal cell carcinoma [76], glioblastoma [77,78], and melanoma [79,80].

FAK inhibitors are an issue of active research [81]. Defactinib is one of the most promising drugs in this regard (see below).

Hypothetically, we may say that cells require permanent and specific signaling from the correct ECM in order to stay alive. Normal cells seem to be continuously monitoring their microenvironment. If this signaling is absent or is incorrect, the cell undergoes apoptosis with a different triggering mechanism, which is called anoikis (detachment-induced apoptosis). Cancer cells can inhibit or modify the vital need for this signaling, and this is called resistance to anoikis. This resistance means that the cancer cell can survive despite the loss of ECM signaling. Once a cancer cell reaches this status, the road for epithelial–mesenchymal transition, invasion, and metastasis is wide open. This explains the importance of anoikis and the role of resistance to anoikis [7,24,82,83,84,85].

Furthermore, not every cell can undergo anoikis. Only attachment-dependent cells are susceptible to anoikis. When they are detached from the epithelium, attachment-dependent normal cells will die. On the other hand, when cancer cells are detached from the epithelium, they resist anoikis and survive. This means that cancer cells develop mechanisms to survive, despite the lack of stimulation and signaling that comes from the extracellular matrix and from fellow epithelial cells. Anoikis resistance is a mechanism to resist apoptosis. It is mainly found in cancer cells and is a necessary step in the progression towards motility, invasion, and metastasis. When anoikis resistance is absent, the cancer cell is unable to progress towards metastasis. This is why anoikis has become an important issue in cancer research.

### 5.3. Integrin-Linked Kinase (ILK)

ILK is a kinase with multifunctional characteristics that regulates integrin and growth factor receptor signaling [86]. It acts as a transduction protein and a scaffolding structure. It is preferentially located at the cell membrane and is a component of focal adhesions. ILK has been found to play a role in cancer progression, and it is a therapeutic target [87]. Despite its name, it is not clear if ILK is really a kinase [88]. ILK has been found to bind the intracellular portion of integrins, and its signaling suppresses anoikis [89,90]. Notwithstanding its multiple pro-tumoral functions, it is not clear if it represents another link between integrin and focal adhesions with anti-apoptotic proteins.

### 5.4. SRC

SRC was identified in the cell in 1976 as the equivalent counterpart of the transforming gene of the avian *Rous sarcoma* virus, v-src, discovered by Rous in 1911. His initial observations describe it as a filterable agent, because viruses were not known at that time [91]. This was the first viral oncogene to be discovered and gave rise to the concepts of proto-oncogene, oncogenes, and tumor suppressor genes.

The SRC protein is a tyrosine kinase signaling protein that specializes in messages that control the growth of cells. It is located inside cells next to the cell membrane. Its main function consists of passing on signals from various protein receptors to intracellular proteins related to growth, survival, cell division, and motility. Regarding FAK, one of SRC’s “clients”, it adds phosphate groups to FAK, further increasing its activity [92,93]. SRC is activated by phosphorylation of Tyr 530, and it can phosphorylate several Tyr residues of FAK [94], as shown in Figure 3.

SRC-mediated phosphorylation of FAK is necessary to couple actin to focal adhesion and adhesion dynamics to survival signaling [95]. SRC has three domains: SH3, SH2, and kinase (SH1). There is also an SH4 domain in some members of the SRC family. SH2 and SH3 are the protein binding regions, while SH1 is the tyrosine kinase region that has an auto-phosphorylation site required for activation.

### 5.5. p130Cas

p130Cas, also known as breast cancer anti-estrogen resistance 1 (BCAR1), is an adaptor protein that interacts with several proteins at the focal adhesion intracellular part. It has a regulatory role in migration and apoptosis [96]. In addition to its interaction with FAK and SRC [97,98], it also interacts with caspase 3 [99] and growth factor receptors [100].

### 5.6. Paxillin

Paxillin: Paxillin is another multifunctional protein at the focal adhesion site and acts as an adapter protein. It recruits other proteins and signaling molecules involved in cell movement and migration [101], as shown in Figure 3.

## 6. Molecular Mechanisms of Anoikis

**Figure 4:**
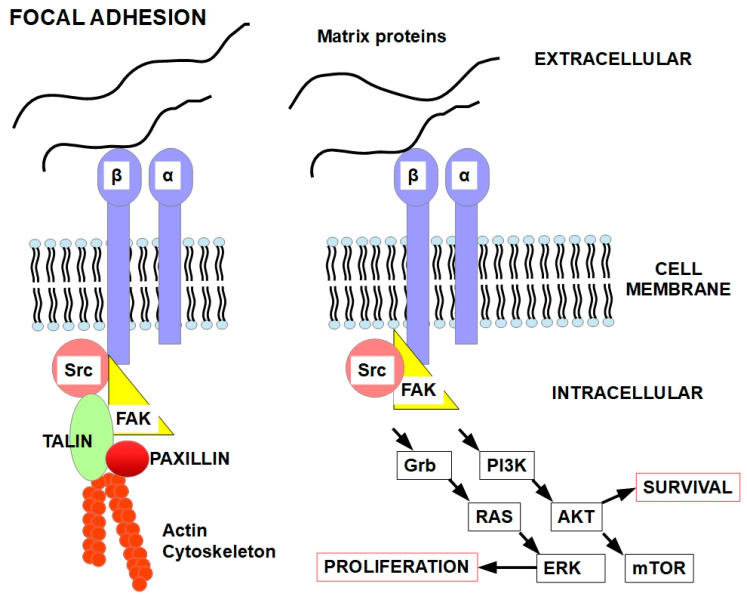
Focal adhesions at the cell membrane concentrate integrins that act mechanically to bind the actin cytoskeleton to the ECM. Importantly, focal adhesions are also signaling hubs that permit the crosstalk between the cell and the ECM. The heterodimer formed by integrins α and β is activated by the matrix proteins. Integrins transmit this activation to various intracellular pathways [107,108]. This keeps the cell “alive” and impedes anoikis [109]. While the cell is attached in its right place, it is the integrins and, additionally, growth factor receptor signaling that maintain cell viability. The FAK-SRC complex activates several signaling pathways, such as the pro-survival PI3K/Akt pathway (right panel) [110]. Microtubule-deregulating agents can disrupt the focal adhesions, leading to loss of adhesion and anoikis [111].

Integrins regulate cell viability and other intracellular features by integrating extracellular conditions with specific intracellular pathways [104]. Integrins also regulate the activity of many cell proteins, such as growth factor receptors, intracellular kinases such as FAK (focal adhesion kinase) and SRC (proto-oncogene tyrosine-protein kinase Src), the cytoskeleton, and other proteins [105,106]. Matrix proteins and/or ligands can activate the heterodimeric integrins to induce intracellular events such as cytoskeletal actin polymerization or signaling towards intracellular pathways. These pathways and intracellular organization are essential for cell viability while attached to the appropriate matrix, as shown in Figure 4.

In 2018, the Nomenclature Committee on Cell Death [112] defined anoikis as a specific form of intrinsic apoptosis triggered by integrin-dependent anchorage deficiency (intrinsic apoptosis is activated by internal signals and involves the mitochondria, while extrinsic apoptosis is apoptosis triggered by external signals such as binding to death receptors on the cell surface [113]).

Anoikis is a triggering mechanism that ends in extrinsic apoptosis and secondarily leads to intrinsic apoptosis. There is evidence showing that the development of resistance to anoikis favors the development of cancers [114], epithelial–mesenchymal transition [115], and metastasis [116].

**Figure 5:**
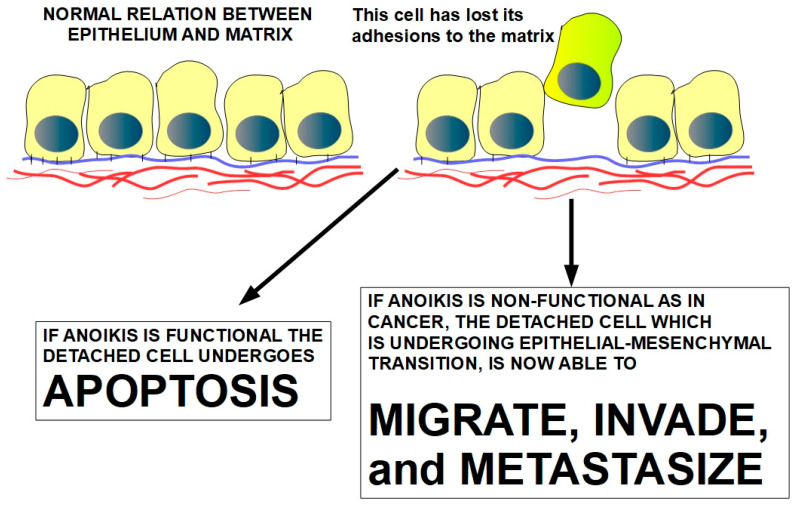
ECM detachment in tumors precedes the abnormal dissemination of cells that leads to metastasis [121]. The drawing shows the destiny of a cell when anoikis is functional and when it is not. Under normal conditions, epithelial cells’ attachment to the matrix keeps them viable; otherwise, they die [24,122]. This drawing shows that, in addition to the genetic regulation of the cell itself, the microenvironment plays an important role in cell growth, differentiation, and cell fate.

Integrin signaling is not the only pathway that prevents anoikis. Growth factor receptors also play a role in this [117,118,119,120]. Below, we discuss mechanisms of suppression of anoikis, as shown in Figure 5.

Anoikis uses both the intrinsic and extrinsic apoptosis pathways. Therefore, it is not a mechanism that is different from other death pathways. What differs from classic apoptosis is the trigger mechanism. Anoikis can use different pathways leading to cell death, but they all end up triggering apoptosis, one way or another. We can consider anoikis as a special form of apoptosis triggered by epithelial or endothelial cell detachment from the matrix. Since it is integrins that connect the cell with its matrix, these transmembrane proteins are the key players in anoikis and are probably also important in resistance to anoikis.

## 7. Specificity of Molecular Interactions

### 7.1. Specificity of the Surface to Which the Cell Is Attached

Meredith et al. [123] showed that some types of cells need to be attached to specific surfaces to rescue them from anoikis. The paper by Meredith et al. has a title that fully depicts the relationship between ECM and cell: “The Extracellular Matrix as a Cell Survival Factor”.

Integrins exhibit ligand specificity determined by their α and β subunit combinations, recognizing distinct extracellular matrix (ECM) components like fibronectin, collagen, laminin, and vitronectin in the tumor microenvironment [124].

### 7.2. Specificity of Integrins

Integrins related to anoikis prevention are also quite specific for different cell types [11,125,126]. This specificity is not found in all cells. Non-epithelial cells do not show any of the specificities mentioned above [127].

- α5β1 specifically binds fibronectin, supporting persistent migration and proliferation via EGFR/AKT signaling in fibronectin-rich tumor matrices. It also plays a role in promoting angiogenesis [128]. α5β1 is up-regulated in hepatocellular carcinoma (HCC), non-small-cell lung carcinoma (NSCLC), and melanoma [23,129]. Furthermore, integrin α5β1 is involved in anoikis resistance or drug resistance of cancer cells [130].
- αvβ3 targets RGD motifs in FN, vitronectin, and fibrinogen, promoting lamellipodia formation, invasion, and angiogenesis through MMP-2 activation.
- αvβ6 binds latency-associated peptide (LAP) of TGF-β and FN, which drive EMT and metastasis in breast cancer [131,132].
- β1 integrins were found to be essential in tamoxifen-resistant breast cancer cells for migration and epithelial–mesenchymal transition that was induced by signals from cancer associated fibroblasts [133].
- In stiff ECMs, β1 integrins (e.g., α2β1 for collagen) sense rigidity, activating FAK-YAP/TAZ to confer anoikis resistance during detachment. β3 integrins compensate for β1 loss, sustaining TGF-β-induced EMT and CTC survival. These shifts enable tumor adaptation to heterogeneous TME stiffness and composition [134].

## 8. Relation Between Cell Detachment and the Apoptosis Pathway

In non-malignant cells, cell surface death receptors are activated by death ligands. These receptors recruit death proteins that form the DISC (death-induced signaling complex), which, in turn, cleaves caspase 8, which is the initiator caspase. Cleaved caspase 8, in turn, cleaves the executor caspases, leading to cell breakdown. FLIP (FLICE-inhibitory protein) is the main inhibitor of the death receptors pathway by preventing caspase 8 activation.

**Figure 6:**
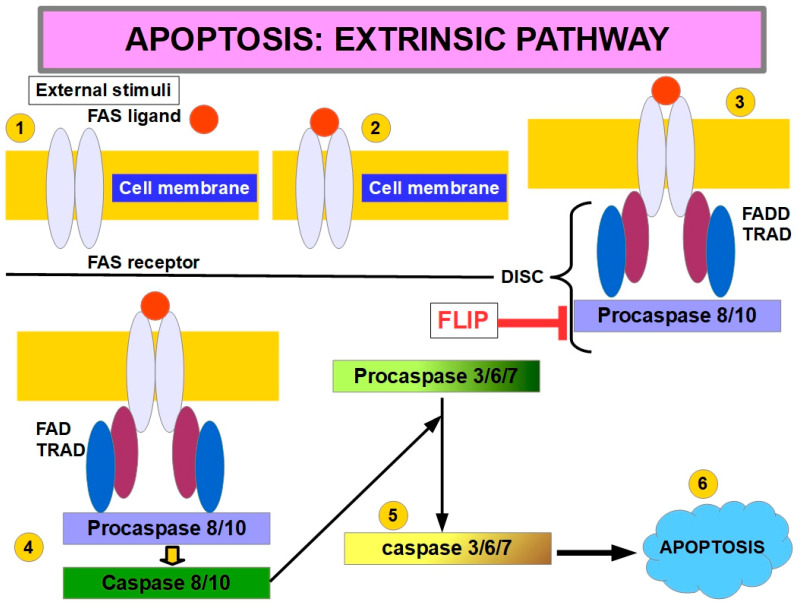
Apoptosis extrinsic pathway. The usual extrinsic pathway is shown. ① cell membrane with death receptor, ② external ligand binding to the death receptor is necessary to activate the pathway through DISC formation ③. DISC: Death-inducing signaling complex procaspase 8 is initially activated to caspase 8 ④, which is an initiator caspase. It activates executioner caspases like 3, 6, and 7 ⑤. Once these are activated, they will degrade the cell ⑥. FLIP is a key regulator of procaspase 8 activation. In anoikis, the lack of integrin signaling leads to an increased number of death receptors, and decreased FLIP leads to caspase 8 activation without the need for an extracellular ligand. The extrinsic pathways seem to predominate as the main pathway in anoikis. Experiments have shown that cell death is prevented when caspase 3 and caspase 8 are inhibited. Caspase 9 inhibition does not achieve the same result [137].

When the cell loses contact with the ECM, death receptors and death receptor ligands are up-regulated and FLIP is down-regulated [135], leading to caspase 8 activation. However, anoikis can occur even without participation of the death receptor: unligated β integrins tails can recruit caspase 8 to the membrane without FADD (Fas-associated protein with death domain) intervention. There, caspase 8 is activated in a death-receptor-independent manner [136], as shown in Figure 6.

**Figure 7:**
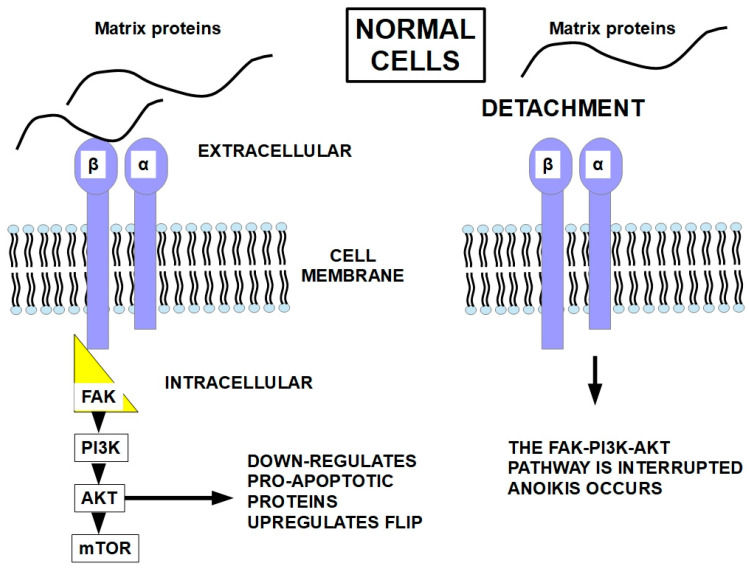
Cell detachment from the matrix interrupts the pathway that represses apoptosis [25,139,140,141]. Integrins continuously regulate cell viability through their interaction with the ECM. Integrins are mechanosensors of forces arising from the matrix, and through their linked pathways, they convert these stimuli to chemically transduced signals [142]. Activating FAK (focal adhesive kinase) and AKT can prevent anoikis [143,144,145].

Matrix attachment seems to protect cells from Fas-receptor-induced apoptosis (Fas is a death receptor on the cell surface), and the opposite occurs with matrix detachment. The interruption of the integrin signaling that follows the integrin-FAK-PI3K-AKT pathway seems to be what increases the activity of the death receptors and decreases FLIP expression [138], as shown in Figure 7.

Activation of caspase 8 is the central event of extrinsic apoptosis. This, in turn, can initiate the intrinsic apoptosis through cleavage of BID releasing t-BID (truncated BID), which unleashes mitochondrial outer membrane permeabilization with cytochrome C release. This is the key in intrinsic apoptosis.

**Figure 8:**
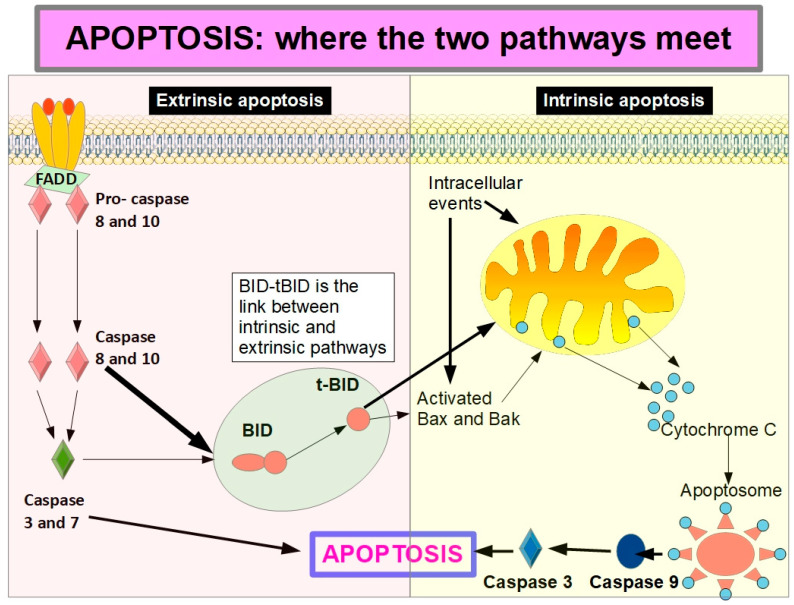
Truncated BID (t-BID) is the connecting protein between extrinsic and intrinsic apoptosis. Truncated BID is generated by the action of caspase 8 (or caspase 10). It can then translocate to the mitochondria, where it promotes outer membrane permeability, leading to cytochrome C release and activation of downstream caspases. This triggers apoptosome assembly, which activates caspases that lead to apoptosis.

Figure 8 shows how the extrinsic apoptosis initiated by caspases 8 activation continues through the intrinsic pathway by cleavage of BID to t-BID (truncated BID). Caspase 8 can cleave BID and thus increases the apoptotic activity reinforced by the intrinsic pathway. However, it has been shown that full-length BID can achieve similar results without being cleaved to t-BID [146].

## 9. Anoikis Resistance Pathways

Resistance to anoikis is essential for tumor development. The malignant cell needs to detach and separate from the ECM during the epithelial–mesenchymal transition process, conferring migratory, invasive, and metastatic abilities. This progression can only take place when the risk of undergoing anoikis is neutralized. Therefore, malignant cells need to develop one or more mechanisms to prevent anoikis. Anoikis resistance (AR) is a possible therapeutic target that is under intensive investigation. Unfortunately, the exact molecular players and their sequence in anoikis resistance are only partially known. For efficient AR, both apoptotic pathways (intrinsic and extrinsic) need to be inhibited. Evidence shows that cancer cells can efficiently abrogate both.

Let us examine the known facts.

### 9.1. Resistance to Anoikis at the Intrinsic Apoptotic Pathway

Apoptosis or anti-apoptosis is the result of the balance between pro- and anti- apoptotic proteins. Pro- and anti- apoptotic proteins can heterodimerize, and it is believed that they can interfere with each other’s functions. Anti-apoptotic proteins can bind BH3 domains of the apoptotic proteins, inhibiting their effects [147,148]. Therefore, the increased expression of anti-apoptotic proteins will result in resistance to apoptosis [149]. Proteins such as Mcl-1, Cav-1, Bcl-x_L_, and 14-3-3ζ are suppressors of anoikis, and their up-regulation induces anoikis resistance in cancer cells [150].

- There is evidence that the expression of the anti-apoptotic members of the Bcl2 family induces increased anoikis resistance [151].
- RAS activation prevents down-regulation of anti-apoptotic proteins during detachment [152].
- SRC activation induces anti-apoptotic protein expression and resistance to anoikis [153].
- According to Woods et al. [154], the anti-apoptotic protein Mcl1 is targeted for proteasomal degradation, and this along with up-regulation of BIM are the initiators of anoikis. Mcl1 ubiquitination and degradation do not occur in malignant cells.

### 9.2. Resistance to Anoikis at the Extrinsic Apoptotic Pathway

- FLIP is the natural inhibitor of the extrinsic pathway. It is a protein with remarkable similarities to caspase 8 and that has a higher affinity to bind DISC, thus replacing caspase 8. This prevents caspase 8 activation at the DISC [155]. FLIP over-expression has been clearly identified as one of the main causes of AR [156,157,158,159].
- While normal cells down-regulate FLIP expression after detachment, malignant cells do not.
- Majwi et al. [160] have shown that inhibiting FLIP at a post-transcriptional level induced anoikis in AR cells when they were detached but did not while they were attached.

## 10. Drivers of Anoikis Resistance

AR is the result of multifactorial changes. Although there is no direct experimental proof, it seems that not one, but many alterations are necessary to induce AR. An indirect proof of this concept is the variety of multiple genetic signatures found in AR in different tumors. These different AR factors are interrelated at some point, forming what we may call an AR network. Parts of this network are discussed below.

### 10.1. Major Drivers

#### 10.1.1. Altered Integrin Expression

Altered integrin expression: Cancer cells down-regulate integrins that promote apoptosis upon detachment (e.g., α5β1) and up-regulate those that support survival (e.g., αvβ3, α6β4). Integrin switch is linked to EMT, which enhances migratory and invasive capabilities while reducing dependence on ECM attachment. Integrin switch has been identified as a cause of AR in melanoma and other tumors. When melanoma invades the dermis, it switches from the normal αvβ1 integrin to αvβ3, which facilitates the acquisition of anoikis resistance [161].

Glioma usually over-express αvβ5 and αvβ3 [162]; breast [163], prostate, and ovarian cancers usually over-express αvβ3 [164]; cervical and colon cancer usually over-express αvβ6 [165]; melanoma usually over-express αvβ3, and α5β1; and squamous cell carcinoma usually over-express αvβ5, and αvβ6 [166].

#### 10.1.2. Activation of Survival Pathways Such as

FAK-SRC pathway. FAK and Src kinases are often activated by switched integrins, promoting cell survival and motility.

EGFR/Ras signaling cooperates with integrins to preserve epithelial architecture and resist anoikis [167,168,169].

PI3K/Akt pathway is a survival pathway that increases anti-apoptotic protein expression [170,171].

#### 10.1.3. Metabolic Reprogramming

Malignant tumors rewire their metabolic landscape by adopting a high-glycolytic-flux phenotype, in which glycolysis and oxphos metabolism coexist, even in the presence of an adequate amount of oxygen (the Warburg effect). This shift supports ATP production (through oxphos) and biosynthesis (through glycolysis, which provides building blocks) under stress, helping cells survive in suspension [172]. Metabolic reprogramming enables cancer cells to resist anoikis by adapting their energy production, redox balance, and biosynthetic pathways to survive without extracellular matrix (ECM) attachment. Enhanced glycolysis and glutaminolysis, increased fatty acid oxidation, and increased pentose phosphate pathway and mitochondrial oxidative phosphorylation support anoikis resistance [173].

Proteomic studies have shown distinct metabolic profiles between adherent and suspended cancer cells, revealing up-regulation of survival-promoting pathways [162]. However, the molecular mechanisms involved have not been clearly established in all cases.

ENOX2 (Ecto-NOX disulfide-thiol exchanger 2) is a cell surface protein involved in redox regulation and cell growth control. ENOX2 is frequently over-expressed in malignant cells [174]. During ECM detachment, ENOX2 supports antioxidant defenses, preventing oxidative-stress-induced apoptosis. Although the role of ENOX2 in anoikis resistance has not been fully proved, we believe that by enabling survival in suspension, ENOX2 facilitates the dissemination of cancer cells through the bloodstream. An indirect proof is that cells expressing increased ENOX2 have a higher metastatic potential [175].

**Figure 9:**
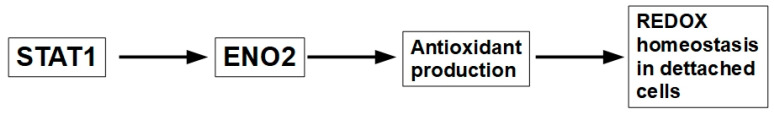
The ENO2 pathway increases detached cell survival by improving redox homeostasis.

Enolase 2 (ENO2). Over-expression of Eno2 has been found to be an important player in anoikis resistance by keeping the redox balance in detached cells, as shown in Figure 9.

Glycolysis and glycolytic enzymes are increased in detached cells [176,177]. By enhancing glycolysis, ENO2 ensures energy supply in anchorage-independent conditions. ENO2 expression correlates with increased migration and invasion, suggesting its role in facilitating metastasis through anoikis resistance. ENO2 over-expression as a cause of AR was initially found in anaplastic thyroid cancers [178], but recently, it has also been found in other tumors as well [179], as shown in Figure 9.

NADPH oxidase 4 (NOX4). NOX4 expression and reactive oxygen species (ROS) generation are up-regulated in suspension gastric cell cultures compared with adherent cultures. Silencing of NOX4 decreases ROS generation and down-regulates EGFR, sensitizing cells to anoikis [180].

#### 10.1.4. Autophagy

Cells recycle components to survive nutrient deprivation during detachment. It is a protective autophagy, although under certain circumstances it can lead to cell death [181,182,183]. Although there is not a clear line that separates the autophagy cell death from protective autophagy, it is presumed that in AR, the role is protective, although we have no firm experimental proof in this regard.

**Figure 10:**
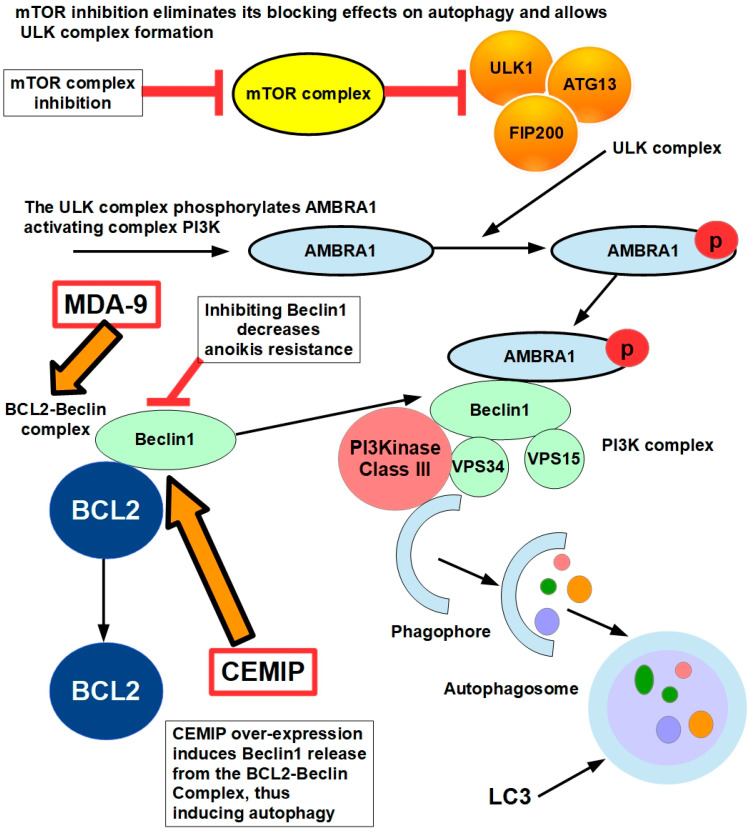
The drawing shows the steps of autophagy. Beclin1, a key component of autophagy initiation, is released from the BCL2–Beclin complex by over-expression of CEMIP permitting the formation of the PI3K complex that induces autophagosome formation. CEMIP amplifies during cell detachment from the extracellular matrix, which induces protective autophagy. Melanoma-differentiation-associated gene-9/Syntenin (MDA-9/Syntenin) is critical for maintaining protective autophagy in anoikis-resistant glioma stem cells [186]. These processes are mediated by modifying FAK and PKC signaling. When MDA-9 is absent, this protective mechanism is disrupted, leading to elevated autophagy and decreased cell survival. Suppressing MDA-9 leads to autophagic cell death and reverses anoikis resistance. Inhibition of autophagy has been shown to reduce anoikis resistance and metastasis. For instance, silencing BECLIN1 and ATG5 genes, which are involved in autophagy, decreased pulmonary metastasis in hepatocellular carcinoma in mice by attenuating anoikis resistance [187].

Yu et al. [184] found that the over-expression of the cell-migration-inducing protein (CEMIP) in detaching cells resulted in protective autophagy by inducing the dissociation of the B-cell lymphoma-2 (Bcl-2)/Beclin1 complex. The Bcl-2/Beclin1 complex regulates the balance between autophagy and apoptosis, playing a critical role in cancer cell survival and resistance to therapy. The Bcl-2/Beclin1 complex forms when Bcl-2 binds to Beclin1 via its BH3 domain, inhibiting Beclin1’s ability to promote autophagy [185], as shown in Figure 10.

When Beclin1 is released, autophagy initiates. Bcl-2 also inhibits apoptosis by sequestering pro-apoptotic proteins like Bax and Bak.

The balanced dual role of Bcl-2 in suppressing both autophagy and apoptosis makes it a central survival factor in cancer.

Cell-migration-inducing protein (CEMIP), also known as KIAA1199, plays a significant role in promoting anoikis resistance, especially in prostate cancer cells, often by enhancing protective autophagy [184]. CEMIP amplifies during cell detachment from the extracellular matrix, which induces protective autophagy. This process involves the dissociation of the B-cell lymphoma-2 (Bcl-2)/Beclin1 complex. Actin transcription factor 4 (ATF4) triggers CEMIP transcription and enhances protein kinase C alpha (PKCα) membrane translocation. This, in turn, regulates the phosphorylation of Bcl-2 at serine70, leading to the dissociation of the Bcl-2/Beclin1 complex and subsequent autophagy.

CEMIP over-expression, triggered by the AMPK/GSK3β/β-catenin cascade, can promote migration and invasion in anoikis-resistant prostate cancer cells by enhancing metabolic reprogramming. This leads to increased pyruvate production, lactate generation, and ATP levels while also impairing detachment-induced apoptosis. Knocking out CEMIP in these cells reverses these effects by reducing the expression of matrix metalloproteinase 2 (MMP2), VEGF, pyruvate dehydrogenase kinase isoform 4 (PDK4), and lactate dehydrogenase A [188].

CEMIP’s role in cancer metastasis includes contributing to cancer cells’ ability to withstand cell death, such as by anoikis and hypoxia [189]. Inhibiting CEMIP-mediated protective autophagy is being explored as a potential therapeutic strategy for metastatic prostate cancer.

#### 10.1.5. Cytoskeleton Reorganization

Cancer cells evade anoikis through dynamic changes in their cytoskeleton. These changes affect cell mechanics, signaling, and interactions with the microenvironment [190]. Cytoskeleton reorganization includes the following:

- Actin remodeling that supports anchorage-independent growth and facilitates migration through tissues;
- Microtubule stabilization that maintains intracellular transport and polarity in detached cells and promotes the formation of survival-promoting structures like giant unilamellar vacuoles, which buffer mechanical stress;
- Intermediate filaments such as vimentin are up-regulated during epithelial–mesenchymal transition (EMT), contributing to structural integrity and resistance to mechanical stress;
- Activation of survival pathways such as the Hippo pathway, particularly YAP/TAZ transcription factors, which promote cell survival and proliferation in detached conditions. Cell detachment activates the Hippo pathway kinases Lats1/2 and leads to YAP phosphorylation and inhibition. This detachment-induced YAP inactivation is essential for anoikis in non-malignant cells, whereas in cancer cells, the deregulation of the Hippo pathway inhibits anoikis. Furthermore, knockdown of YAP and TAZ restores anoikis [191].

#### 10.1.6. Epithelial–Mesenchymal Transition (EMT)

EMT is a biological process where epithelial cells lose their polarity and adhesion properties, acquiring mesenchymal traits like motility and invasiveness. This transformation is tightly linked to anoikis resistance in cancer progression [192].

Down-regulation of E-cadherin in EMT disrupts epithelial junctions, allowing cells to detach. This detachment normally triggers anoikis, but EMT suppresses apoptotic signaling [193]. EMT activates the PI3K/Akt, MAPK/ERK, and NF-κB pathways, which inhibit apoptosis and promote survival in detached conditions. These pathways also support anchorage-independent growth and therapy resistance. We suggest that anoikis resistance development is a necessary and integral part of the EMT process. Both phenomena are so intertwined and interdependent that it is not easy to establish a clear dividing line. For example, EMT involves remodeling of actin and intermediate filaments (e.g., vimentin), enhancing cell motility and structural integrity during detachment. Another example is EMT increasing the population of cancer stem-like cells, which are inherently resistant to anoikis and contribute to recurrence and metastasis. E-cadherin and ankyrin-G are lost coordinately during EMT, conferring anoikis resistance. Finally, targeting EMT regulators or restoring epithelial traits may sensitize tumors to anoikis and reduce metastasis.

### 10.2. Other Drivers of Anoikis Resistance

#### 10.2.1. Extracellular Acidity

It is a well-known fact that extracellular acidity, a constant hallmark of cancer [194], is an important facilitator of metastasis. What is less known is that one of the mechanisms involved in this facilitation is an increased resistance to anoikis. The exact molecular steps that lead from extracellular acidity to increased anoikis resistance are not well known. On a speculative basis, but based on some experimental evidence, we may assume that extracellular acidity increases anoikis resistance by stimulating autophagy [195]. mTORC1 and NF-kB signaling seem to have a role in resistance to anoikis in the acidic extracellular matrix [196].

Acidic conditions in the tumor microenvironment promote anoikis resistance by triggering protective autophagy through AMPK/mTOR activation and suppressing miR-3663-3p. This enables cancer cells to survive detachment. The adaptation supports metastasis in acidic niches like HCC and melanoma, where low pH (e.g., 6.7) up-regulates survival pathways without altering proliferation in attached cells. This acidic adaptation leads to increased cloning efficiency, migratory ability, and survival of melanoma cells in non-adherent conditions and within the bloodstream. Overall, pH is recognized as one of the specific factors driving anoikis resistance in cancer cells.

#### 10.2.2. Intracellular Alkalinity

Intracellular alkalinity, (pH > 7.2) promotes cancer cell survival during detachment from the extracellular matrix, enhancing anoikis resistance. This reverse pH gradient (alkaline intracellular pH with acidic extracellular pH) supports glycolytic shifts, proliferation, and adaptation to hypoxic conditions in tumor cells [197,198]. An alkaline intracellular pH drives the Warburg effect by boosting glucose uptake and reducing oxygen consumption. This favors cancer cell proliferation over normal cells. Enzymes like GAPDH and GPI show pH-dependent activity, where alkaline conditions optimize glycolytic flux critical for detached cell survival. Acidifying pHi selectively impairs cancer metabolism, reversing these adaptations and exposing vulnerabilities.

Anoikis-resistant cells maintain alkaline pHi via proton transporters (e.g., Na^+^/H^+^ exchangers, MCTs), which activate glycolytic enzymes like PFK-1 and sustain energy during matrix detachment. Repeated detachment–reattachment cycles induce adaptive anoikis resistance with transcriptional shifts toward oxidative phosphorylation and reduced apoptosis, paralleling in vivo ascites-derived cells. This adaptation heightens metastasis, chemoresistance (e.g., to paclitaxel), and immune evasion via down-regulated MHC-I [199,200].

In summary, high intracellular pH promotes anoikis resistance in cancer cells by optimizing glycolytic enzyme activity, sustaining ATP production, and enabling metabolic adaptations during extracellular matrix detachment.

#### 10.2.3. V-ATPase Pump Up-Regulation

Up-regulation of these proton exporters seem to promote anoikis resistance [201]. We believe that this is due to their contribution to extracellular acidity and intracellular alkalinity.

#### 10.2.4. Nitric Oxide (NO) and Caveolin-1

Chanvorachote et al. [202] found that NO can impair the apoptotic function of lung carcinoma cells after detachment by inhibiting the ubiquitin-proteasomal degradation of Caveolin-1. Caveolin-1 (Cav-1) promotes anoikis resistance in cancer cells by stabilizing anti-apoptotic proteins like Mcl-1 and activating survival pathways such as Src/EGFR/ITGB1, PI3K/Akt, and MEK/ERK, facilitating anchorage-independent growth and metastasis. Over-expression correlates with aggressive phenotypes in lung, gastric, and breast cancers, where it links ECM detachment cues to evasion of apoptosis [203,204,205].

#### 10.2.5. Reactive Oxygen Species (ROS) and Growth Factor Receptors

NADPH oxidase 4 (NOX4) expression and ROS generation are up-regulated in some tumors such as gastric cancer, leading to anoikis resistance by inducing EGFR activation [180].

#### 10.2.6. EWS/FLI Oncogenic Protein

Ewing sarcoma is an aggressive pediatric bone and soft tissue cancer that is characterized at the molecular level by the presence of a chromosomal translocation: t(11; 22) (q24; q12). The encoded chimeric oncoprotein from this translocation is known as EWS/FLI, which is the result of the fusion of the amino-terminal domain of EWS to the carboxyl-terminus of FLI [206]. This protein has been shown to play a key role in anoikis resistance, because when the protein was down-regulated, anoikis followed [207]. This protein is not found in other tumors.

#### 10.2.7. Oncoviruses and Anoikis Resistance

Certain viruses such as HPV, EBV, and HBV can promote cancer development by increasing anoikis resistance in infected cells, further supporting the link between anoikis resistance and cancer progression [208,209].

#### 10.2.8. Mir141-Sp1 Axis

**Figure 11:**
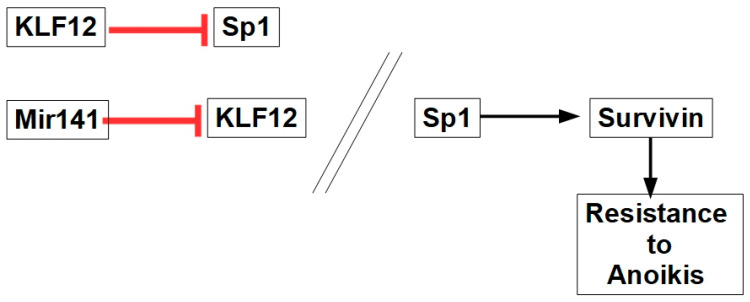
The role of MiR141 and Sp1 in anoikis resistance. KLF12 expression and activity are barriers to anoikis resistance.

MicroRNA141 expression is increased in some tumors such as ovarian cancer [210]. Mir141 targets the KLF12 (Kruppel-like factor 12) protein, which inhibits Sp1 (specificity protein 1). Sp1 is a transcription factor that promotes survivin transcription, which increases anoikis resistance, as shown in Figure 11.

#### 10.2.9. NHE1 (Sodium Hydrogen Exchanger 1)

NHE1 plays a role in anoikis resistance, although this has not been firmly proven. Pedersen [211] offered a hypothesis of the mechanism: “…*it is well-established that NHE1 is an important effector downstream from integrin activation and necessary for integrin-dependent cell spreading. It is, thus, tempting to speculate that the loss of anchorage-dependence seen in many cancer cells could be related to the elevated NHE1 activity and/or altered NHE1-dependent signaling in these cells, circumventing the requirement for cell adhesion for survival*”.

It is also known that apoptotic enzymes require an acidic intracellular pH [177,212,213,214]. One of the main functions of NHE1, albeit not the only one, is proton extrusion through the cell membrane, thus preventing intracellular acidification [215,216]. NHE1 is one of the main players in maintaining an alkaline intracellular milieu. The effects of intracellular alkalinization on anoikis resistance have been discussed above. NHE1 maintains intracellular alkalinity by extruding protons in exchange for sodium ions, counteracting acidosis during cellular stress like detachment or apoptosis. This pH homeostasis supports survival signaling, volume regulation, and resistance to anoikis in cancer cells by activating pathways such as Akt via ERM scaffold interactions [217].

#### 10.2.10. FER Kinase (Feline Sarcoma-Related Kinase)

FER kinase is a non-receptor cytoplasmic tyrosine kinase [218] that controls migration and metastasis of invasive human breast cancer cell lines by regulating α_6_- and β_1_-integrin-dependent adhesion [219]. By enhancing integrin signaling, FER helps cells maintain survival signals even when detached from the ECM. FER kinase activity has also been identified in AR in other tumors such as ovarian, lung, and prostate cancers [220,221,222,223]. FER kinase can activate the PI3K/Akt and MAPK/ERK pathways, suppressing apoptosis and supporting anchorage-independent growth.

#### 10.2.11. Epigenetic Factors

DNA methylation and histone modifications alter gene expression to favor survival. These changes can silence pro-apoptotic genes and activate anti-apoptotic ones [224].

#### 10.2.12. Loss of E Cadherin

Loss of E cadherin is a characteristic feature of EMT. Cells with lost expression of E cadherin show a higher rate of metastasis and increased resistance to anoikis [225].

Table 1 summarizes the anoikis resistance concepts.

## 11. Anoikis Resistance in Pancreatic Cancer

Anoikis resistance in pancreatic cancer is a crucial factor in its progression and metastasis, allowing cancer cells to survive detachment from the extracellular matrix. Several intertwined and overlapping mechanisms contribute to this resistance. However, one mechanism is considered the main promoter of anoikis resistance in pancreatic cancer: over-expression and activation of the transcription factor STAT3. STAT3 activation is achieved by increased phosphorylation at Tyr705, leading to enhanced expression of anti-apoptotic proteins like Bcl-2 and Mcl-1 and increased migratory, invasive, and metastatic potential of cancer cells. Inhibiting STAT3 reduces anoikis resistance and tumor formation in pancreatic cancer models, highlighting its vital role in cancer progression and metastasis.

### 11.1. Anoikis Resistance Drivers in Pancreatic Cancer

The following AR drivers have been identified in PDAC.

#### 11.1.1. The PI3K/AKT Pathway Activation

**Figure 12:**
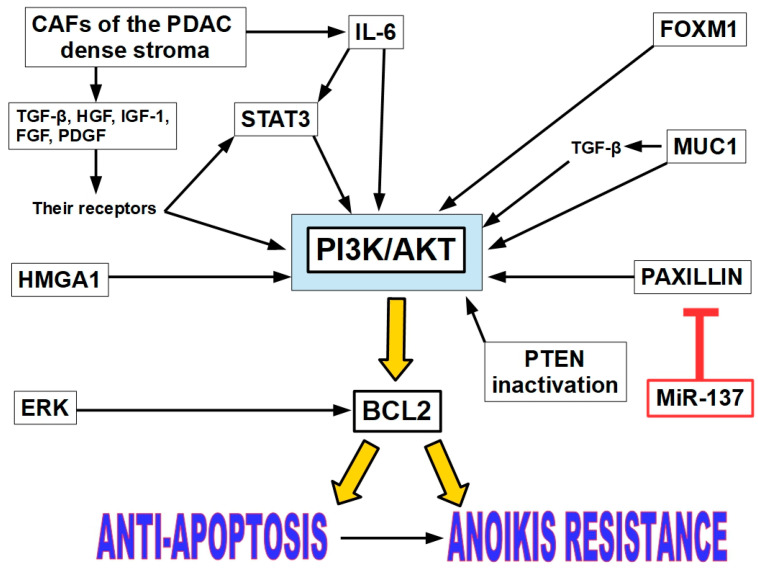
STAT3 is considered the main driver of anoikis resistance. However, its effects on AR are mediated through the PI3K/AKT pathway. This is also the case with most of the AR players, with the exception of ERK.

This pathway promotes cell survival, proliferation, angiogenesis, and resistance to apoptosis. We believe that the activation of this pathway represents the central core of AR in PDAC (pancreatic ductal adenocarcinoma) [226], as shown in Figure 12. PI3K/AKT is often hyper-activated in PDAC due to mutations in KRAS, PTEN loss, or over-expression of growth factor receptors.

Chronic inflammation, one of the PDAC predisposing factors, is induced by various mechanisms and plays crucial roles in PDAC development. Cytokines, chemokines, and growth factors present in the PDAC desmoplastic environment activate the PI3K/AKT pathway [227]. This is a clearly pro-survival pathway that induces the expression of the anti-apoptotic proteins of the BCL2 family.

PI3K (a lipid kinase) phosphorylates PIP2 to generate PIP3, which recruits AKT to the plasma membrane, where it is activated by PDK1 and PDK2. Activated AKT phosphorylates multiple downstream targets that inhibit apoptosis [228,229]. For example, AKT phosphorylates and inactivates BAD, a pro-apoptotic Bcl-2 family member. It also inhibits caspase 9, a key initiator of the intrinsic apoptotic pathway [230]. Furthermore, AKT enhances the activity of transcription factors like NF-κB, which up-regulate anti-apoptotic genes such as Bcl-2 and Bcl-xL [231].

Blocking the PI3K/AKT pathway led to a decreased metastatic burden in mouse models and cell lines by lowering the rate of circulating cancer cells [232].

#### 11.1.2. PI3K/AKT Pathway Activators

The PI3K/AKT pathway can be activated by many receptors and proteins.

Growth factors and cytokines produced by CAFs. Cancer-associated fibroblasts (CAFs) of the PDAC desmoplastic stroma produce growth factors such as TGF-β, HGF, FGF, PDGF, and IGF-1 [233,234,235,236], which bind to their receptors, which, in turn, initiate signaling pathways that can activate PI3K/AKT. TGF-β is a potent activator of the PI3K/AKT pathway via TRAF6-dependent mechanisms, especially in cancer and fibrotic conditions [237,238]. FGF binding its cognate receptors can also activate the PI3K/AKT pathway [239]. The same situation happens with IGF1 [240] and PDGF [241].

STAT3 activation. Signal transducer and activator of transcription 3 (STAT3) plays a critical role in conferring anoikis resistance to pancreatic cancer cells. Cells that resist anoikis show increased expression and phosphorylation of STAT3 at Tyr 705, which also enhances their migratory and invasive characteristics. Interleukin-6 (IL-6) treatment can enhance anoikis resistance by phosphorylating STAT3, while STAT3 inhibitors like piperlongumine can induce anoikis and prevent tumor formation and metastasis in pancreatic cancer models [242]. STAT3 can be activated by IL-6 as an upstream effector. IL6 can simultaneously activate both the JAK/STAT3 and PI3K/AKT pathways. This dual activation is crucial for inflammatory and survival responses in cells [243]. Furthermore, CAFs produce IL-6 in pancreatic cancer [244]. In PTEN-deficient cancer cells, inhibition of the PI3K/AKT pathway can lead to feedback activation of STAT3, which then limits the efficacy of PI3K-targeted therapies. STAT3 can up-regulate genes that encode components or regulators of the PI3K/AKT pathway, thereby indirectly enhancing its activity. In cells transformed by mutant PI3K, STAT3 becomes phosphorylated and contributes to oncogenic transformation. Blocking STAT3 impairs PI3K-driven tumorigenesis. STAT3 induces anoikis resistance in PDAC lines (AsPC-1, Panc-1, L3.6PL), with 65–75% of cells surviving anchorage-independent conditions and gaining 2–2.5-fold higher invasion/migration. STAT3 inhibition sensitizes cells to anoikis and reduces metastatic potential [84]. Probably for this reason, many authors consider STAT3 as the main driver of AR [84,242,245,246].

MUC1 (mucin 1). MUC1 is a transmembrane glycoprotein commonly over-expressed and aberrantly glycosylated in many cancers, particularly in PDAC. Its cytoplasmic tail interacts with key signaling molecules, including components of the PI3K/AKT pathway. The cytoplasmic tail of MUC1 can bind to PI3K, facilitating its activation and subsequent phosphorylation of AKT. Furthermore, MUC1 enhances the stability and activity of growth factor receptors, amplifying PI3K/AKT signaling cascades [247]. Targeting tumor-associated MUC1 with agents like TAB004 has shown promise in reducing colony-forming potential and triggering anoikis in pancreatic ductal adenocarcinoma (PDA) cells, suggesting its potential to curb tumor relapse, prevent metastasis, and enhance chemotherapy efficacy [248]. Furthermore, MUC1 over-expression can induce TGF-β signaling in PDAC [249].

HMGA1. Over-expression of HMGA1 (High-Mobility Group A1) promotes anoikis resistance in pancreatic adenocarcinoma cells through a PI3-K/Akt-dependent mechanism, leading to increased Akt phosphorylation and kinase activity and reduced caspase 3 activation [250].

PTEN inactivation: virus-induced protein APOBEC3G. APOBEC3G can induce AR by inactivating PTEN and subsequently activating Akt kinase [251].

PAXILLIN. MicroRNA-137 (miR-137) is down-regulated in pancreatic cancer and promotes anoikis by modulating the AKT signaling pathway, with paxillin (PXN) identified as a target of miR-137 that promotes AKT activation [252].

FOXM1 (Forkhead Box M1) regulation. FOXM1, a transcription factor, is identified as a differentially regulated gene in pancreatic cancer cells, and its expression is controlled by platelets. Manipulating FOXM1 expression affects platelet-mediated anoikis resistance, suggesting it as a potential therapeutic target [253]. FOXM1 plays a pivotal role in regulating cell cycle progression, DNA repair, and tumorigenesis. Its interaction with the PI3K/AKT signaling pathway is especially significant in cancer biology. FOXM1 can transcriptionally up-regulate genes like *RacGAP1*, which, in turn, activates the PI3K/AKT pathway [254]. This leads to enhanced cell proliferation, migration, and invasion, particularly in cervical cancer cells. The PI3K/AKT pathway can also regulate FOXM1 expression. AKT activation promotes FOXM1 nuclear localization and transcriptional activity, creating a positive feedback loop. FOXM1 and FOXO3 influence sensitivity to AKT inhibitors. FOXM1 over-expression can reduce responsiveness to these therapies [255].

#### 11.1.3. ERK/BCL Pathway

Activation of a signaling pathway involving ERK and subsequent over-expression of BCL-2 can confer resistance to anoikis in pancreatic cancer cells [256]. This pathway seems to be independent of the PI3K/AKT pathway. The ERK-BCL2 pathway links extracellular signals to anti-apoptotic responses, promoting cell survival and contributing to cancer progression. ERK activation is triggered by growth factors, cytokines, or oncogenic signals via the RAS/RAF/MEK cascade. ERK1/2 signaling promotes cell survival by activating pro-survival BCL2 proteins and repressing pro-death proteins [257].

Figure 12 resumes the intertwining and overlapping factors that play a role in AR in PDAC.

#### 11.1.4. STAT3 as an Independent Driver of AR

Figure 12 and the preceding considerations show STATAR effects mediated through the PI3K/AKT pathway. However, STAT3 has important independent effects. STAT3 can directly promote the expression of anti-apoptotic and survival genes. In some pancreatic cancer models, STAT3-driven survival is found even when PI3K/AKT is not chronically activated. PI3K/AKT signaling can enhance STAT3 activity, reinforcing survival signals.

STAT3 activation induces V-ATPase pump expression and activity, aiding cancer cells in managing intracellular pH and ROS levels, further promoting resistance to anoikis [258]. In addition, STAT3 also positively regulates the expression of another proton extruder: NHE3 (sodium/hydrogen exchanger 3) through interaction with Sp1 and Sp3 (specificity protein 1 and 3). Sp1, Sp3, and STAT3 bind cooperatively to the NHE3 promoter [259]. The relationship with NHE1 is not that clear in pancreatic cancer. In gastric cancer, STAT3 has been found to translocate to the nucleus after activation and binds to promoter regions of target genes, including SLC9A1, which encodes NHE1. Up-regulation of NHE1 occurs in gastric cancer cells is resistant to 5-FU. In this case, STAT3 activation leads to increased NHE1 expression, which helps maintain intracellular pH and supports cell survival under stress [260].

STAT3 can cooperate with HIF-1α to enhance carbonic anhydrase IX (CAIX) gene transcription, especially in hypoxic tumor microenvironments [261]. CAIX helps cancer cells survive acidic stress by regulating intracellular and extracellular pH, a process enhanced by STAT3 signaling.

Through V-ATPase, CAIX and NHE3, and probably NHE1, STAT3 modulates a whole setting of proton extruders with the ability to maintain a high intracellular pH that hinders apoptosis.

In melanoma and pancreatic cancer cells, silencing STAT3 leads to 70–80% reduction in anoikis resistance. It also reduces migration and invasion.

STAT3 also influences the expression of EMT-related proteins and signaling pathways that promote cell survival in suspension, contributing to aggressive cancer phenotypes through anoikis resistance [262,263,264]. STAT3 has been found to be a genetic modifier of TGF-beta-induced EMT in KRAS mutant pancreatic cancer cells. D’Amico et al. [265] showed that STAT3 activation conferred increased KRAS-dependency to PDAC cells.

PDAC is a particularly hypoxic tumor, where hypoxia-inducible factors (HIFs) play an important pro-tumoral role. Adaptation to hypoxia is a necessary and essential role for pancreatic cancer cell survival. STAT3 and HIF-1α cooperate to regulate gene expression under hypoxic conditions, enhancing tumor survival, angiogenesis, and metabolic adaptation. STAT3 enhances HIF-1α’s transcriptional activity by stabilizing its expression and facilitating its nuclear localization [266,267,268].

This interaction forms part of an autocrine loop where STAT3 and HIF1α mutually regulate each other to sustain cancer cell survival, growth, and adaptation under hypoxic stress [269].

#### 11.1.5. Genetic Signature of Anoikis Resistance in PDAC

Anoikis-related gene signature: A multi-omics study identified seven prognostic genes (MET, DYNLL2, CDK1, TNFSF10, PIP5K1C, MSLN, GKN1) linked to pancreatic adenocarcinoma prognosis and anoikis. Their related proteins, such as EGFR and MMP2, also significantly impact prognosis. These genes were found to be highly expressed in pancreatic cancer cell lines compared to normal pancreatic cells [270].

Mutations: Mutations in KRAS, P53, and CDKN2A have a significant impact on the prognosis of pancreatic adenocarcinoma, highlighting their role in the tumor microenvironment and potentially anoikis resistance. For example, mutant KRAS continuously activates downstream pathways like PI3K/AKT and RAF/MEK/ERK, which suppress apoptotic signals. KRAS mutant cells shift toward glycolysis and glutamine metabolism, supporting survival in anchorage-independent conditions.

The integrin and ephrin receptor families were up-regulated in all PDAC samples, irrespective of outcome, supporting an important role of the interaction between pancreatic cancer cells and the surrounding desmoplastic reaction in tumorigenesis and cancer progression [271].

Figure 12 shows that AR in PDAC is a multifactorial phenomenon, as it is also in other tumors. One or other factors may be more important in specific tumors at a specific moment, but probably one factor alone would not be enough to support AR. This issue has not been experimentally proven yet. In pancreatic cancer, it is evident that different AR players yield to create an AR phenotype. We must also underline that in PDAC the desmoplastic environment is a contributor to AR.

Crosstalk and redundancy of AR molecules and pathways contribute to reinforcing malignant cell survival, and inhibiting one pathway can be compensated by others [272].

## 12. Anoikis and EMT Relationship

During EMT, cells down-regulate epithelial markers and up-regulate mesenchymal genes, enabling detachment, migration, and resistance to anoikis. Key traits include the following:

(1) CDH1 (E-cadherin gene), EPCAM, and occludin down-regulation, thus facilitating detachment.
(2) Up-regulating VIM (vimentin gene, supporting cytoskeleton reorganization), CDH2 (N-cadherin, which replaces E-cadherin), SNAI1 (Snail, which represses E-cadherin), TWIST1/2 (promotes mesenchymal gene expression and stemness), and ZEB1 (increases migratory abilities).

Vimentin promotes anoikis resistance by enhancing survival signaling and facilitating cell clustering in detached cells. Both mechanisms are absolutely necessary for successful metastasis. Vimentin binds to the internal pool of beta1 integrins, protecting them from lysosomal degradation. Cell detachment induces vimentin phosphorylation at its Ser38 residue, which induces the release of beta 1 integrins and their translocation to the cell surface. Beta 1 increased surface expression, in turn, induces cell clustering and activates the PI3K/AKT survival pathway, thereby evading anoikis [273,274]. Vimentin has also other AR actions, such as:

- Interacting with the 14-3-3 protein, preventing its availability for the pro-apoptotic cascade [275];
- Up-regulating pro-survival molecules like NF-kB [276];
- Activating the Ras/Raf/Erk proliferative pathway;
- Activating autophagy to prevent apoptosis [277,278].

The interaction between 14-3-3 and vimentin contributes to cell motility, especially in contexts like EMT and cancer metastasis. Vimentin’s reorganization, supported by 14-3-3, enables cells to adapt to new environments and resist anoikis [279,280].

**Figure 13:**
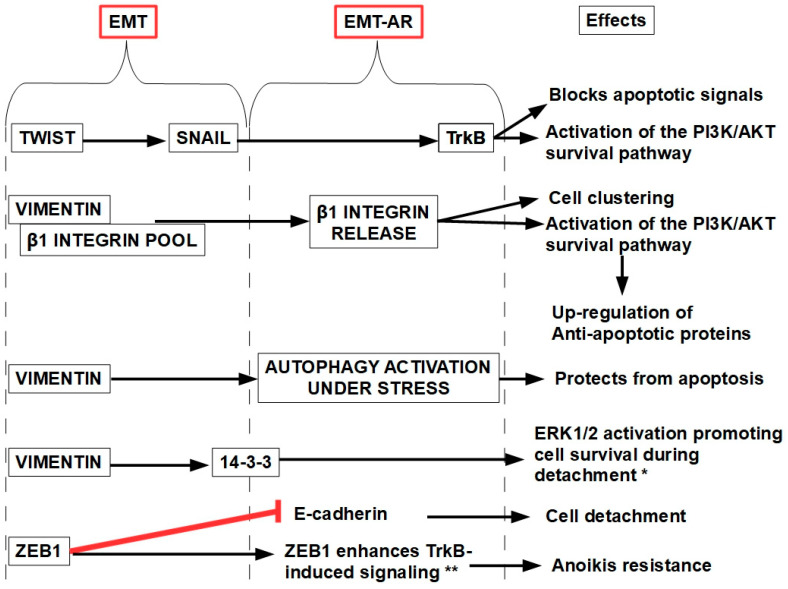
A snapshot showing the multiple relations between EMT and AR. * [282]; ** [283]. Targeting EMT-related proteins has been shown to restore anoikis sensitivity. ZEB1 suppression restores E-cadherin expression and sensitizes cells to anoikis. Snail and Twist inhibitors reverse EMT, promoting cell–cell adhesion and apoptosis upon detachment.

Smit et al. [281] described a Twist–Snail–TrkB axis, which represents a clear relation between two EMT-related genes like Twist and Snail with an AR-related gene like TrkB (see below), as shown in Figure 13.

Netrin-1 is another up-regulated protein during EMT, supporting survival signaling. Netrin-1 has an anti-apoptotic effect by binding its receptor UNC5H [284]. NP137 is a monoclonal antibody against netrin-1 and inhibits EMT, increasing epithelial cell populations, reducing metastasis, down-regulating anoikis resistance, and enhancing chemotherapy response [285].

The transcription factor Sp1 (specificity protein 1), which binds GC-rich promoter regions and regulates genes involved in cell survival, proliferation, and stress response, is another link between EMT and AR. Sp1 promotes resistance to anoikis and drives epithelial–mesenchymal transition (EMT) by regulating key survival and migration-related genes, contributing to cancer progression and metastasis [286]. Sp1 has the following functions:

- Activates survival pathways: Sp1 up-regulates components of the PI3K/Akt, MAPK, and JAK/STAT pathways, which suppress apoptosis triggered by ECM detachment [287].
- Enhances anti-apoptotic gene expression: It promotes transcription of Bcl-2, Survivin, and other anti-apoptotic proteins that help cells evade anoikis [288,289].
- Supports anchorage-independent growth: By maintaining survival signals, Sp1 enables cancer cells to thrive in suspension, a key step in metastasis.
- Sp1 can increase intracellular pH: Intracellular alkalinity is a handicap for the apoptotic process. Sp1 can alkalinize the cell by increasing proton export through the promotion of NHE1, NHE2, and NHE3 [290].
- Sp1 represses epithelial markers, such as E-cadherin, weakening cell–cell adhesion.
- Activates mesenchymal genes: It promotes expression of vimentin, fibronectin, and N-cadherin, facilitating cytoskeletal remodeling and migration. Sp1 directly regulates the transcription of the vimentin gene by binding to its promoter [291].
- Cooperates with EMT transcription factors: Sp1 interacts with Snail, ZEB1, and Twist, amplifying EMT signaling and enhancing resistance to anoikis [292].
- Sp1 regulates Beclin1 expression by binding to its promoter, influencing autophagy and cancer cell survival [293,294].
- Sp1 regulates integrin gene expression, influencing cell adhesion, migration, and signaling (including several β1 and α subunits of integrins) [295,296,297].

All the data mentioned above hints towards a unified integral idea of EMT and AR as part of the same indivisible process.

## 13. Anoikis and Inflammatory Signaling

Inflammatory signaling promotes anoikis resistance in cancer cells primarily by activating survival pathways like NF-κB, which up-regulates anti-apoptotic proteins and cytokines such as IL-6 and TNF-α. Cytokines including IL-17A enhance migration, cytoskeletal remodeling via Rho GTPases, and direct suppression of apoptosis, enabling detached cells to survive circulation as CTCs. This creates a pro-metastatic microenvironment with increased angiogenesis and immune evasion.

NF-κB activation, initiated by TNF-α or by mechanical stress in detached cells, up-regulates IAPs (such as XIAP, cIAP1/2) through RhoA/ROCK-mediated cytoskeletal tension, thereby inhibiting caspase-dependent anoikis. IL-6 from CAFs or stressed cells reinforces this through JAK/STAT3, which promotes YAP/TAZ activity and metabolic adaptation under detachment. TNF-α in prostate cancer drives anoikis resistance by sustaining survival signals in the inflammatory milieu [298]. NF-ĸB modulates cell death mechanisms, notably by inhibiting apoptosis and ferroptosis [299].

IL-17A enhances anoikis resistance in TNBC cells by activating Src, RhoA/Cdc42, and COX-2, thereby promoting migration without altering proliferation, and by up-regulating TNF-α, IL-1β, and chemokines such as CXCL2/CCL20. In vivo, IL-17A blockade reduces CTC survival and tumor angiogenesis (CD34 expression), shifting immunity toward TGF-β1 dominance and fewer CD8+ cells [300]. In breast cancer, IL-17A sustains CTCs against shear stress and anoikis via inflammatory cascades. NF-κB in colitis-associated colorectal cancer amplifies IL-6/TNF-α loops, fostering anoikis evasion during EMT. Mechanical confinement in metastasis further links inflammation to NF-κB-driven IAPs, enhancing immune escape [301].

In metastatic osteosarcoma cells, a pro-inflammatory pathway has been identified that promotes anoikis resistance: the IL-8/C-X-C chemokine receptor (CXCR) 1/Akt-signaling pathway. IL-8 treatment of osteosarcoma metastatic cells increased anoikis resistance in vitro [302].

On the other hand, CXCL12 chemokine expression and secretion was found to increase anoikis in colorectal carcinoma cells by activating Bim-mediated intrinsic apoptosis [303].

In summary, IL-6, TNF-α, IL-17A, and CXCL8 (IL-8) emerge as the cytokines most strongly linked to promoting anoikis resistance in tumor cells, activating survival pathways like NF-κB, STAT3, and AKT/ERK.

## 14. Anoikis Resistance and ECM Stiffness

Normal cells monitor their respective cellular microenvironment. Regarding cell fate, the main inputs of anoikis are related to the surveillance and interaction of cell–ECM contacts. Changes in this interaction have direct effects on intracellular signaling cascades. For example, PDAC has a particularly stiff ECM, and the over-expression of FAK is directly proportional to the aggressiveness of the tumor [63].

ECM stiffness in the tumor microenvironment promotes anoikis resistance by activating mechanosensitive pathways that enhance cell survival and metabolic adaptation during detachment. Stiff matrices precondition cancer cells via integrin clustering and FAK signaling, enabling them to withstand circulation as CTCs [85,304]. Stiffness sensing plays a significant role in anoikis resistance, particularly in cancer progression. The tumor microenvironment contributes to cancer cells’ anoikis resistance by modulating matrix stiffness [305].

Substrate stiffening can activate the mTORC1 pathway via an integrin-GSK3β-FTO axis, leading to increased mTOR levels and anabolic metabolism. This provides a growth advantage on rigid substrates. Conversely, inhibiting this pathway upon ECM detachment enhances autophagy, contributing to tumor cell resilience against anoikis [306]

## 15. Anoikis Resistance Genes and Pathways

During AR implementation, the cell over-expresses a group of genes and pathways, as discussed below.

### 15.1. Genes

- MUC1: This is a big transmembrane glycoprotein, usually found over-expressed in epithelial cancers [307] and particularly in pancreatic cancer. It disrupts cell adhesion and promotes survival signaling, helping cells evade anoikis. MUC1 glycosylation stimulates apoptosis and chemotherapy resistance to drugs such as bortezomib, trastuzumab, and tamoxifen, among others [308,309]. It also promotes multidrug resistance genes. Under stress conditions, MUC1 is cleaved in two molecules, MUC1-N and MUC1-C, which create inward pro-survival signals. The role of MUC1 in cancer goes well beyond anoikis resistance but will not be discussed here (for a review, see Chen et al. [310], Lan et al. [311], and Qing et al. [312]).
- KL (Klotho): The KL gene was identified in 1997 as an anti-aging gene and was initially believed to be a tumor suppressor gene/protein [313]. It is now evident that KL is a controversial gene/protein. Most articles describe KL as a tumor suppressor [314,315], but it also shows pro-tumoral effects that “increases cellular migration, anchorage-independent growth, and anoikis resistance in hepatoma cells” [316,317].
- MNX1 (motor neuron and pancreas homeobox 1): This is a pro-tumoral transcription factor linked to oncogenic transformation and anoikis resistance through metabolic and proliferative pathways. MNX1 was found to play an important role in developing anoikis resistance in glioblastoma. MNX1 expression was higher in more malignant glioma cell lines. MNX1 allowed malignant cells to bypass anoikis while reducing fibronectin adhesion [318]. MNX1-induced anoikis resistance was mediated by activation of tyrosine kinase receptor B (TrkB), which is a downstream effector. It also promotes proliferation by up-regulating cyclin E [319] and CCDC34 (coiled-coil domain-containing 34) [320]. In addition to glioblastoma, MNX1 was identified as an anoikis resistance gene in many tumors such as renal cell carcinoma [321], colon cancer [322], and acute myeloid leukemia [323].
- MMP3 and TIMP1 in laryngeal squamous cell carcinoma have been recognized as anoikis resistance genes [324].
- HMCN1 (Hemicentin 1): This is a gene associated with extracellular matrix remodeling and may facilitate detachment survival. It has been identified among renal cell carcinoma anoikis resistance genes [325] and ovarian cancer [326].
- ADCY10 (Adenylate Cyclase 10): This is involved in cAMP signaling, which can modulate survival pathways under stress. It has been identified as an AR gene signature in lung adenocarcinoma [327].
- TrkB (tropomyosin receptor kinase B) is a key suppressor of anoikis, enabling cancer cells to survive detachment and promoting metastasis. TrkB is a receptor tyrosine kinase that binds brain-derived neurotrophic factor (BDNF). It plays a critical role in neuronal survival and development but is hijacked by cancer cells to evade anoikis. TrkB activation blocks apoptotic signals triggered by loss of cell adhesion, allowing cells to survive detachment [328]. It also activates PI3K/AKT and MAPK/ERK signaling cascades, promoting proliferation and survival [329], promotes epithelial–mesenchymal transition, and increases growth and metastatic potential [330]. TrkB activity has been found to be increased in tumors such as neuroblastoma, breast, lung, pancreatic, gastric, colorectal, ovarian, and cervical cancers [331,332,333,334]. TrkB inhibitors have been developed, as follows:

▪ Experimental selective TrkB Inhibitors

ANA-12: This is a non-competitive TrkB antagonist used in preclinical studies. It inhibits BDNF-induced TrkB activation and downstream signaling.

GNF-5837: This is a small molecule that selectively inhibits TrkA, TrkB, and TrkC, with potent activity against TrkB in vitro.

▪ FDA-approved pan-TrkB inhibitors

Larotrectinib (Vitrakvi): This is highly selective for TRK proteins and is approved for solid tumors with NTRK fusions.

Entrectinib (Rozlytrek): This inhibits TRK, ROS1, and ALK and is approved for *NTRK*-fusion-positive cancers.

TrkB inhibitors have shown promise in preclinical models by restoring anoikis sensitivity and reducing metastasis.

### 15.2. Pathways

- PI3K/AKT and MAPK/ERK signaling: These pathways are frequently activated in anoikis-resistant cells, promoting survival and proliferation.
- Metabolic reprogramming: Cancer cells adapt their metabolism (e.g., increased glycolysis) to survive without matrix attachment.
- EMT (epithelial–mesenchymal transition): EMT-related genes are often up-regulated, enhancing motility and resistance to cell death.

The list of AR-related genes and pathways is incomplete. Only the most important have been mentioned above. Furthermore, different tumors show different AR genetic signatures.

For example, in prostate cancer, the following anoikis resistance genes have been identified: EGF, MYC, PLK1, EZH2, AFP, NOX4, BMP6, and MMP11 [335]. Expression of PLK1 (polo like kinase 1) was the most predictive gene regarding survival and tumor evolution. PLK1 is a cell cycle regulator that is activated by NF-kB during cancer cell detachment and prevents β-catenin degradation [336] and activates STAT3 [337]. PLK1 induces epithelial–mesenchymal transition and cell motility [338]. PLK1 inhibitors such as volasertib are under development [339].

In lung adenocarcinoma, an anoikis score was built based on four genes: TLE1, GLI2, PLK1, and BAK1 [340].

A predictive model was built with three anoikis-related genes for cutaneous melanoma based on FASLG, IGF1, and PIK3R2. Other AR-related genes have also been identified, although they have not been used for predictive models in melanoma: DAPK2, BIRC5, PIK3CG, MMP9, PRKCQ, HAVCR2, SPIB, CDC25C, LCK, SPTA1, and IKZF3.

Jiao et al. [341] evaluated a risk model for breast cancer based on 10 prognostic AR-related genes: CD24, PTK6, NAT1, BRCA2, IKZF3, HMGA1. EDA2R, SPIB, BST2, and NTRK3.

The reason for mentioning these AR gene signatures is to show their heterogeneity in different tumors. Therefore, although anoikis resistance is a universal phenomenon in cell transformation, the way to achieve it shows that there are many different genetic mechanisms.

## 16. Targeting Anoikis Resistance

Pharmacological targeting of resistance to anoikis is a promising therapeutic objective [342]. While no drugs have yet been approved, several are currently in late-stage development. There are also approved drugs that can be repurposed to target AR. Drugs targeting anoikis resistance aim to re-sensitize cancer cells to anoikis. By overcoming anoikis resistance, these drugs can potentially inhibit cancer cell survival, metastasis, and recurrence.

### 16.1. V-ATPase Pump Inhibitors

It has been shown that archazolid, a V-ATPase inhibitor, reduced FLIP expression, increased caspase 8 activity, reduced active integrin-β1, and increased the proapoptotic protein BIM in several types of malignant cells in vitro. This was the initial effect of archazolid; however, later, it could increase anoikis resistance. In vivo, it reduced the number of metastases in mouse breast cancer [343]. Archazolid-treated endothelial cells increased tumor cell adhesion mediated by β1-integrins expressed on the tumor cells [344]. Archazolid induces a pro-adhesive phenotype that seems to counteract AR.

V-ATPase inhibitors have been shown to reduce invasiveness and metastasis in cancer [345,346]. However, V-ATPase inhibitors require individual drug testing and not a generalization. For example, activated lansoprazole reduced cancer cell adhesion to ECM [347].

Digoxin and its derivatives. It has been found that digoxin has important effects promoting apoptosis and reducing anoikis resistance in circulating cancer cells [348] and non-circulating cancer cells [349,350]. This effect is achieved by targeting a protein fraction of the Na^+^/K^+^ ATPase pump. It also sensitizes cancer cells to anoikis by stimulating the proteasomal degradation of the anti-apoptotic protein Mcl1 [351].

### 16.2. Microtubule-Destabilizing Agents

These drugs interfere with the cytoskeleton, disrupting focal adhesions and inducing anoikis [352]. Microtubule-disrupting agents induced paxillin phosphorylation and activated anoikis through the loss of focal adhesion structures. ILK over-expression rescued the antimicrotubule-mediated loss of cell viability.

### 16.3. Signaling Pathways Inhibitors

Curcumol is a bioactive hemiketal sesquiterpenoid obtained from the essential oil of the rhizomes of Curcuma rhizoma. It shows good absorption and tissue distribution after oral administration to rats. Curcumol’s anti-cancer activity can be increased by chemical manipulation [353]. Curcumol is a potent inducer of apoptosis in numerous cancer cells. It targets key signaling pathways such as MAPK/ERK, PI3K/Akt, and NF-κB. It also inhibits cell survival in triple-negative breast cancer (TNBC) by targeting AR through the BIM pathway [354], and it can activate p73 and PUMA [355]. Furthermore, it enhances the sensitivity of doxorubicin in TNBC [356]. Additionally, it has shown pleiotropic anti-cancer effects in many other tumors [357,358].

Fucoxanthinol produces anoikis in colorectal cancer by suppressing integrin signaling [359,360,361,362].

Tunicamycin causes endoplasmic reticulum stress and unfolded protein response due to protein aggregation that leads to anoikis [363].

Thapsigargin is a compound that induces endoplasmic reticulum stress and releases calcium ions from the endoplasmic reticulum into the cytoplasm, which can contribute to anoikis [364,365].

Dasatinib is an FDA-approved tyrosine kinase inhibitor used for the treatment of chronic myeloid leukemia and other hematologic cancers with the Philadelphia chromosome. It inhibits the activity of BCR-ABL kinase and SRC family kinases along with other specific oncogenic kinases, including c-KIT, ephrin receptor (EPH) kinases, and the PDGFß receptor. Importantly, it can induce anoikis through SRC inhibition [366,367,368,369].

Celecoxib is a COX2-selective inhibitor that triggers anoikis in colon carcinoma by targeting protein-Crk-associated substrate 130 kDa (p130Cas), thus deregulating focal adhesions. Celecoxib induces the proteolytic cleavage of p130Cas and the nuclear translocation of the 31 kDa generated fragment, leading to apoptosis [370]. This effect is independent of COX2 inhibition. It was found that celecoxib induces anoikis in osteosarcoma by inhibiting the PI3K/Akt pathway [371]. Similar effects have been found in canine breast cancer cells [372].

Gefitinib is an EGFR inhibitor that induces anoikis in cervical cancer cell lines [373,374].

MEK inhibitors inhibit the MAP kinase pathway downstream of MEK; therefore, there is no phosphorylation of ERK. It has been shown that MEK inhibitors can restore anoikis sensitivity in human breast cancer cells [375] through increased BIM [376]. Selumetinib (AZD6244) is an MEK1 and MEK2 inhibitor that has been found useful for the treatment of neurofibromas [377]. It has also been tested in melanoma metastasis [10].

Disulfiram is used for chronic alcohol dependence and is a drug that has shown many antitumor properties. Among them, disulfiram can induce copper-dependent cell death. Importantly, it induces anoikis in TNBC cells [378].

Metformin can counteract anoikis resistance in medullary thyroid carcinoma, probably through down-regulation of mTOR/6SK and phosphorylated ERK [379].

### 16.4. Integrin Inhibitors

Cilengitide is a selective αv integrin inhibitor that has been tested against glioblastoma [380]. The integrin αvβ3 is involved in angiogenesis, cell migration, and proliferation. It is expressed at low levels in normal cells and over-expressed in glioblastoma, melanoma, breast, prostate, and pancreatic cancer cells [381]. By targeting integrin αvβ3, cilengitide down-regulates the FAK-SRC-AKT pathway in endothelial cells, acting as an angiogenic drug. The mechanism of action seems to be complex. In a first step, the integrin inhibition produces anchorage loss of the endothelial cells, and this, in turn, induces anoikis [382,383,384,385].

However, clinical results with cilengitide in glioblastoma have been disappointing, with no overall survival improvement. For this reason, there has been no further development of the drug [386].

JSM6427 is an α1β3 inhibitor that showed decreased growth in investigative studies to treat glioblastoma [387].

ILKAS is an ILK anti-sense oligonucleotide that has been used in glioblastoma [388].

TDI4161 is a small molecule that has been developed for the treatment of osteoporosis that specifically targets integrin αvβ3. It has not been fully tested in cancer [389].

### 16.5. FAK Inhibitors

Doxycycline. This antibiotic has been found to induce growth inhibition and apoptosis in some tumors. One of the mechanisms involved in these effects is inhibition of FAK phosphorylation [390].

A group of specific FAK inhibitors has been developed. Their use as monotherapy has shown poor results. On the other hand, results have improved substantially when they are used together with MEK inhibitors.

Defactinib is a FAK inhibitor that has undergone encouraging clinical trials in association with the MEK inhibitor avutometinib for the treatment of low-grade ovarian serous cancer, as shown in Table 2.

### 16.6. Repurposed and Nutraceutical Drugs

Many over-the-counter drugs and nutraceuticals have been shown to counteract AR. Among them are the following:

Alpha-mangostin. This is a member of the class of xanthones, isolated from the stems of Cratoxylum cochinchinense. It has antioxidant, antimicrobial, and antitumor activities. Mangostin was found to induce anoikis in anoikis-resistant hepatocarcinoma cells [396].

Aspirin. Aspirin increased sensitivity of breast cancer cells to anoikis, decreasing the number of distant metastases. It also decreased the number of circulating tumor cells in vitro. Thromboxane A2 is increased upon detachment from the ECM, and aspirin decreased thromboxane A2 through COX1 inhibition [397,398,399].

Berberine [400]. The natural pentacyclic triterpenoids betulinic acid (BA) and betulin are found in many trees and plants. White birch tree (*Betula alba*) bark is particularly rich in BA. Many therapeutic effects of these compounds have been described in AIDS, malaria, and cancer, which are probably the most important. BA has clear anti-cancer effects in some tumors, particularly melanoma. However, these benefits are not universal and cannot be taken for granted in all tumors. Probably, they are inducers of apoptosis in ectoderm-originated malignancies such as melanoma and neuroblastoma. However, betulinic acid was found to also induce apoptosis and anoikis in other tumors such as prostate, colon, and breast cancers, among others. BA was found to be an effective inducer of apoptosis in malignant cells [401,402,403,404,405] while preserving normal cells [406].

BA in melanoma. In 1995, Pischa et al. [407] found that BA was a selective inhibitor of human melanoma in vivo. Its main mechanism of action was induction of apoptosis. This was repeatedly confirmed by many further publications of different authors [408,409,410,411,412,413,414,415,416,417]. Furthermore, it has been found that BA had additive or synergistic effects with chemotherapeutic drugs against melanoma [418,419] and with other non-chemotherapeutic drugs such as digoxin [420]. However, there is a publication that found that BA induced increased expression of the anti-apoptotic protein Mcl1 [421]. This seems to contradict all the mentioned findings, and we have no plausible explanation for this result.

Apigenin. Apigenin is a natural component of celery. It has been found to sensitize breast cancer cells to anoikis by a similar mechanism to aspirin [422].

### 16.7. Integrin–EGFR Interaction Inhibitors

Doxazosin is used to treat arterial hypertension and benign prostatic hyperplasia. Doxasosin derivatives have been found to exert important antitumor effects in prostate cancer, and importantly, it was found to reverse anoikis resistance in vitro [423].

### 16.8. STAT3 Inhibitors

Several STAT3 inhibitors with potential therapeutic applications have been identified. Most STAT3 inhibitors work by preventing its dimerization through binding to the SH2 domain, blocking downstream signaling pathways involved in tumor progression and immune suppression. Several are under evaluation for clinical development, with some showing promising experimental results in in vitro and animal models. None has achieved FDA approval.

N4. Of particular interest in pancreatic cancer is a small-molecule inhibitor called N4 that has shown strong antitumor activity by directly targeting STAT3 activation [424]. This inhibitor binds specifically to the SH2 domain of STAT3, suppressing tumor growth, metastasis, and remodeling the tumor microenvironment. It has been tested in vitro and in animal models. Human testing is not available yet.

WB436B. WB436B selectively binds to STAT3, inhibiting Tyr705 phosphorylation. WB436B suppresses pancreatic cancer growth and metastasis in vivo and prolonged survival of mice bearing the tumor [425].

C188-9 (TTI-101). This is an inhibitor evaluated in clinical trials for various cancers designed to suppress STAT3 activity by targeting its SH2 domain. There are preclinical tests in breast cancer showing increased reduction in cancer cell viability, which was superior to that reached with other traditional chemotherapy drugs [426].

OPB-111077. This is a high-affinity inhibitor of STAT3 activity and mitochondrial function, undergoing clinical evaluation [427,428]. A phase I clinical trial [429] in patients with hepatocellular carcinoma showed limited preliminary benefits. There are also other phase I trials with some encouraging results in acute myeloid leukemia in combination with conventional chemotherapeutics [430].

Stattic. This is an earlier-discovered small-molecule inhibitor targeting the STAT3-SH2 domain and inhibiting STAT3 activity. It is used mainly in research and preclinical studies.

YY002. This is a highly selective inhibitor with favorable pharmacokinetics showing promising in vitro and in vivo effects. It is potentially superior to some compounds in clinical development [431].

Ibuprofen. Ibuprofen has been shown to inhibit the STAT3 pathway, but it acts indirectly rather than as a direct STAT3 inhibitor. Ibuprofen reduces both the total STAT3 protein and its phosphorylated form (p-STATY705), particularly under hypoxic conditions in cancer cells, leading to decreased STAT3 activity. This inhibition contributes to reduced cancer cell viability, migration, and inflammation-related gene expression. The mechanism involves ibuprofen’s effect on histone modifications (H3 methylation and acetylation) [432] and suppression of related inflammatory and stemness genes via COX2-dependent pathways. It also down-regulates other transcription factors like NFκB, further impairing STAT3-mediated pathways. However, binding assays indicate that ibuprofen does not directly bind to STAT3 but modulates its expression and activation levels through upstream regulatory mechanisms. This means that while ibuprofen impacts STAT3 activity, it is not a classical direct STAT3 inhibitor like small molecules designed to bind STAT3’s SH2 domain (e.g., N4 or Stattic). Rather, it exerts modulatory effects on STAT3 expression and phosphorylation through upstream pathways like COX2 and epigenetic regulation [433].

Other non-steroidal anti-inflammatory drugs (NSAIDs). Some NSAIDs have shown abilities to inhibit STAT3 in addition to ibuprofen, such as celecoxib, meloxicam, and others [434,435,436,437,438].

### 16.9. Sp1 Transcription Factor Inhibitors

Some NSAIDs such as tolfenamic acid and celecoxib have been shown to down-regulate Sp1 transcription factor.

The drugs that have shown effects against anoikis resistance are summarized in Table 3.

## 17. Discussion

High-turnover epithelial tissues, such as the gastrointestinal mucosa, permanently shed a huge number of cells. These cells need to be eliminated because they would be able to colonize in other tissues, generating eventual neoplastic growth if they have the appropriate mutations. Since the number of cells shed in the order of billions, the risk of finding a few of them with adequate mutations for developing a cancer would be very high. Fortunately, multicellular organisms have developed anoikis as a mechanism that induces the programmed destruction of these cells. Anoikis begins shortly after the cell detaches from the ECM and is shed. Anoikis is simply an apoptotic process, which is triggered by detachment from the ECM. The purpose of anoikis is to prevent detached cells from surviving. Therefore, anoikis is a physiological process playing a relevant role in development and homeostasis. High-mobility cells, such as mesenchymal cells, and naturally non-adherent cells, such as mature hematological cells, are resistant to anoikis. Anoikis is often deregulated in several diseases, such as cancer. Importantly, malignant cells have also developed a mechanism to resist anoikis. Cancer cells are insensitive to anoikis and can survive, migrate, invade, and proliferate without any attachment to the ECM.

Epithelial–mesenchymal transition (EMT) is an early step in transformation. During this process, cells acquire mesenchymal characteristics including morphology and biochemistry. Functional changes include migration and invasion abilities, and for this, anoikis resistance is of capital importance, as otherwise, transformed cells would die as a consequence of anoikis-induced apoptosis [281]. Therefore, the moment for anoikis resistance installation is EMT or close to it. Part of the essential function of EMT in cancer cells is to transform an epithelial attachment-dependent cell into a mesenchymal-like attachment-independent one.

EMT and anoikis resistance are interdependent and probably inter-coordinated processes. Cells that do not develop anoikis resistance cannot leave their ECM attachment, and therefore there is no invasion nor metastasis without anoikis resistance. Anoikis is the first and most important natural barrier to metastasis, while anoikis resistance opens the gates for invasion and distant spread of malignant tumors.

**Heterogeneity of anoikis resistance**

Cancer cells can use different strategies to achieve anoikis resistance, and different tumors usually show different genetic anoikis signatures. In this review, we analyzed the mechanisms governing pancreatic ductal adenocarcinoma development of anoikis resistance in depth. Other tumors show alternative mechanisms; however, shared traits are usually found. For example, almost all the different mechanisms show over-expression of anti-apoptotic proteins, decreased expression of pro-apoptotic proteins, and over-expression of pro-survival pathways, particularly PI3K/AKT and STAT3. Therefore, all the different mechanisms of anoikis resistance converge in inhibiting apoptotic pathways while activating survival signaling. Heterogeneity of anoikis resistance mechanisms represents a problem for a selective pharmacological approach to treat AR.

**Oxidative stress and metabolic reprogramming**

Cancer cells live in an oxidative intracellular milieu, which is cautiously handled to avoid excessive cellular oxidation, leading to oxidative stress. Interestingly, this moderate oxidative load correlates with induction of anoikis resistance. ROS activate SRC kinase that, in turn, activates pro-survival pathways, such as ligand-independent EGFR phosphorylation and anti-apoptotic protein BIM degradation. Moderate oxidative stress, metabolic reprogramming, and hypoxia are partners in supporting anoikis resistance. To these partners we must add extracellular acidity and intracellular alkalinity.

**Intra- and extracellular pH in anoikis resistance**

Cancer cells up-regulate proton-export mechanisms that rapidly remove intracellular protons and acidify the surrounding extracellular matrix. This increased performance is achieved by protein over-expression or by elevated activity of proton extruders such as NHE1, V-ATPase proton pumps, carbonic anhydrase IX and XI, and monocarboxylate transporters 1 and 4. Proton export impedes anoikis by maintaining an alkaline intracellular pH (pHi), which enhances glycolytic flux and promotes cancer cell survival during detachment from the extracellular matrix. Proton exporters like carbonic anhydrase IX (CA-IX) expel H^+^ ions, raising pHi and activating pH-sensitive glycolytic enzymes upstream of pyruvate kinase. This boosts aerobic glycolysis, glucose uptake, and lactate production, providing energy and biosynthetic intermediates that support anoikis resistance [439].

In detached cells, such as during metastasis, elevated proton export enables robust spheroid formation and reduces apoptosis, as seen in CA-IX-over-expressing breast cancer models. Neutralizing extracellular acidity diminishes this effect, lowering metastatic potential. Carbonic anhydrase IX (CA-IX) stands out as the proton exporter most strongly linked to anoikis resistance in cancer cells. Other notable exporters include Na^+^/H^+^ exchangers (NHE1) and V-ATPase, which maintain pH gradients, favoring survival during metastasis by expelling protons extracellularly [440].

Cancer cells are characterized by extracellular acidity and intracellular alkalinity. This inversion of the normal pH gradient is the ideal condition for AR because apoptosis-related enzymes require an acidic cytoplasm. Therefore, intracellular alkalinity is a barrier to apoptosis.

Intracellular acidification can trigger apoptosis by activating specific proteases and disrupting cellular homeostasis [212,441,442,443]. This process plays a critical role in programmed cell death across various biological contexts. We believe that alkalinization of intracellular pH plays an important role in achieving anoikis resistance. Proton extruders sustain metabolic flux in anchorage-independent conditions, reducing apoptosis in models like breast and gastric cancers. Inhibiting them disrupts this resistance, demonstrating the therapeutic potential of this approach [444].

Tumor intracellular pH research has been mainly focused on NHE1 effects and their inhibition; however, there is evidence showing that the isoform NHE7 can be an important player in anchorage-independent growth [445]. NHE7 is one of the nine membered NHE gene families that regulate the luminal pH of the Golgi apparatus and endosomes.

V-ATPase inhibitors also sensitize cancer cells to anoikis by disrupting proton export, leading to intracellular acidification, increased ROS production, and accumulation of misfolded proteins [446,447,448]. Inhibitors like bafilomycin A and concanamycin A enhance anoikis susceptibility in suspended cells from cervical, breast, and melanoma lines by elevating ROS levels and impairing protein folding, which promotes apoptosis over survival. This effect is more pronounced in detached conditions, reducing multicellular aggregate formation [446,447,448].

**EMT and AR are different faces of the same process**

In this paper, we have reviewed the convincing relationship between EMT and AR in some detail. We believe that they are both part of one integrated and indivisible process and suggest that this relationship can be exploited in developing treatments for AR. A good example in this regard is that miRNA200c is able to regulate EMT and AR simultaneously, and miRNA200c can restore anoikis sensitivity by acting through repression of genes related to EMT [449].

**ROS and anoikis resistance**

ROS promote anoikis resistance in cancer cells by activating survival pathways like SRC-EGFR signaling, which sustains metabolic adaptation and prevents apoptosis during matrix detachment. Low shear stress in circulating tumor cells further boosts ROS-NO to stabilize Caveolin-1, suppressing ubiquitination and apoptosis. Mitochondrial complex III and NADPH oxidases (NOX4) serve as the primary source of ROS during matrix detachment in cancer cells [450,451,452].

However, ROS have a dual role. Moderate ROS levels confer resistance, while excessive ROS trigger anoikis through mitochondrial damage and protein misfolding [453]

**Targeting anoikis resistance**

Anoikis resistance represents a viable therapeutic target, although effective clinical strategies have yet to be realized. There are many possible targets, such as integrins, kinases like FAK and SRC, and pathways such as PI3K/AKT and STAT3, among others. If we were to define AR in a very simplified manner, we will say that anoikis resistance is controlled by constitutive activation of FAK. Thus, FAK should be considered a primary target for inhibiting AR.

Although targeting anoikis resistance is a promising therapeutic strategy under active research, it remains a challenging objective. Many drugs have been shown to induce anoikis in tests; however, no truly effective pharmaceutical has yet been developed. Defactinib, a specific FAK inhibitor, showed encouraging results in low-grade serous ovarian cancer when administered with MEK inhibitors such as avutometinib. However, many other drugs have failed initial promise, with cilengitide being the best example. Unfortunately, currently there is no ideal drug to prevent anoikis resistance.

## 18. Conclusions

Normal cells die upon detachment from the ECM through an apoptotic mechanism that usually starts as extrinsic apoptosis and finally involves the intrinsic apoptotic pathway as well. This is known as anoikis. Cell detachment from the ECM is a major event in normal cells’ lives because it inevitably leads to death through anoikis. On the other hand, cancer cells develop resistance to anoikis, which permits invasion and metastasis. To survive ECM detachment, cancer cells initiate multiple mechanisms that prevent their death.

Anoikis has long been an overlooked area of study. However, it has now emerged as a leading subject of study. A simple phrase defines this interest: there is no metastasis without anoikis resistance. Intensive research on anoikis is not more than twenty years old, and many advances have been made; however, the most important one is still lacking: how to prevent/inhibit anoikis resistance. There has been an accelerated development of small molecules that target essential kinases. We hope that anti-FAK and anti-SRC molecules will be able to modulate AR in the future. miRNA-based therapies are also on the verge of revolutionizing cancer treatment. In this regard, miRNA200c can be an interesting tool that modulates AR. Anoikis is a complex process that is in the middle of other processes such as EMT, apoptosis, and cell migration. For anoikis to occur, there is an intensive crosstalk between many molecules and pathways. The same is true for resistance to anoikis. The characters in the anoikis resistance drama are almost all identified. This is the first step. The second step, modulating them, is still missing. We also believe that a breakthrough in targeting anoikis resistance is close.

## 19. Future Perspectives

Pharmacological targeting of anoikis resistance using bioactive compounds and small-molecule inhibitors shows promise in overcoming tumor migration and invasion in cancers such as gastric cancer and hepatocellular tumors. Nanomedicine innovations, such as platelet-based carriers and nano-enzymes, aim to enhance delivery and specificity, addressing bioavailability challenges in clinical translation. Combination therapies incorporating FAK inhibitors such as defactinib are being investigated for their potential to reverse EMT and re-sensitize cancer cells to apoptosis. Anoikis-related gene signatures enable robust prognostic models, predicting survival in cancers, such as lung, gastrointestinal, bladder, and head/neck squamous cell carcinoma. These models integrate with immune infiltration and drug sensitivity analyses to guide personalized treatments and identify high-risk patients early. Future efforts prioritize biomarker-driven trials for metastasis prognosis and therapy response.

Investigations into cytoskeleton-signaling interfaces, translational regulation, and tumor cell–blood interactions will deepen mechanistic understanding of anoikis evasion. Applications extend to non-cancer fields like stem cell engineering, tissue repair, and cardiovascular diseases, where anoikis regulation influences cell fate and therapy efficacy. Single-cell sequencing and CRISPR screens are poised to uncover novel regulators, including non-coding RNAs.

## Figures and Tables

**Table 1:** Other drivers of anoikis resistance.

Driver	Mechanism
Extracellular acidity	Stimulates autophagy via AMPK/mTOR activation and down-regulates miR-3663-3p.
Intracellular alkalinity	Promotes cell survival, supporting glycolytic switch, which maintains energy requirements.
V-ATPase pump up-regulation	Increases extracellular acidity and increases intracellular alkalinity.
Nitric oxide	Inhibits the ubiquitin-proteasomal degradation of Caveolin-1.
Increased ROS	Induces EGFR activation.
EWS/FLI oncogenic protein	Only found in bone. The mechanism has not been fully elucidated.
Oncoviruses	HPV, EBV, and HBV can sequester anoikis resistance mechanisms for their survival.
Mir141-Sp1 axis	Mir141 inhibits Sp1 inhibitor KLF12, releasing Sp1, which up-regulates survivin.
NHE1	Increases intracellular pH and decreases extracellular pH.
FER Kinase	FER boosts integrin signaling, enabling cell survival without ECM attachment.
Epigenetic factors	Silences pro-apoptotic genes and activates anti-apoptotic ones.
Loss of E. cadherin	Promotes anoikis resistance by disrupting cell–cell adhesion, activating survival signaling like PI3K/Akt and Src, and enabling anchorage-independent growth critical for metastasis.

**Table 2:** Clinical trials associating defactinib with other drug(s).

Pathology	Trial Data	Associated Drug	Stage/Results
Diffuse type gastric cancer	NCT06487221		Enrolling patients
Pancreatic ductal adenocarcinoma	[391]	Pembrolizumab and gemcitabine	Phase I. 15 patients. 7% partial response, 53% stable disease, 40% disease progression.
Refractory ovarian cancer	NCT01778803 [392]	Paclitaxel	Phase I/Ib. 18 patients. 1 complete response, 1 partial response, 1 stable disease.
Kras mutant non-small-cell lung cancer	[393]	Defactinib monotherapy	Phase II. 55 patients. 1 patient with partial response
Pleural mesothelioma	[394]	Defactinib monotherapy	Phase II. 173 patients treated with 171 controls. There were no overall survival benefits.
Low-grade ovarian serous cancer	[395]	Defactinib with avutometinib	Response of 45% with the association and 10% with avutometinib monotherapy. Tumor shrinkage was observed in the vast majority of patients on the combination and monotherapy arms, with 86% and 90%, respectively.

FDA breakthrough therapy designation was given to avutometinib + defactinib in recurrent low-grade serous ovarian cancer.

**Table 3:** Drugs that act on anoikis resistance and their target site.

Targeting Anoikis Resistance		
Action	Drugs	Target
V-ATPase proton pump inhibitors	Omeprazole Lansoprazole Pantoprazole Others. Digoxin	Cell membrane proton pump
Microtubule-destabilizing agents	Paclitaxel Docetaxel	Mitotic apparatus
Signaling pathway inhibitors	Curcumol	MAPK/ERK, PI3K/Akt, and NF-Κb, BIM, PUMA
	Fucoxanthinol	Integrin signaling
	Tunicamycin	ER stress and UPR
	Dasatinib	BCR-ABL kinase and SRC family kinases
	Thapsigargin	ER stress
	Celecoxib	PI3K/Akt pathway and p130Cas
	Gefitinib	EGFR
	MEK inhibitors	MEK
	Disulfiram	Cuproptosis
	Metformin	mTOR/6SK and phosphorylated ERK
Integrin inhibitors	Cilengitide	Integrin αvβ3
	JSM6427	Integrin α1β3
	ILKAS	ILK
	TDI4161	Integrin αvβ3
FAK inhibitors	Doxicyclyne	FAK phosphorylation/activation
	Defactinib	FAK phosphorylation/activation
Repurposed and	Aspirin	Thromboxane A2
nutraceuticals	Berberine	Apoptotic proteins
	Apigenin	Thromboxane A2
Integrin–EGFR inhibitors	Doxazosin	Integrin–EGFR interaction
	Digoxin	Alpha subunit of the Na^+^/K^+^ ATPase pump
STAT 3	N4	STAT3 pathway
inhibitors	C188-9	STAT3 pathway
	OPB-111077	STAT3 pathway
	Stattic	STAT3 pathway
	YY002	STAT3 pathway
	Ibuprofen	STAT3 pathway
	NSAIDs	STAT3 pathway
Sp1	Tolfenamic acid	Sp1 expression/effects
inhibitors	Celecoxib	Sp1 expression/effects

## Data Availability

No new data were created or analyzed in this study. Data sharing is not applicable to this article.
